# Hypothyroidism and Thyroxine-Induced Hyperthyroidism Differentially Affect Cathepsins, Mct8, and Thyroid Tissue Architecture in Male Mice

**DOI:** 10.3390/ijms27209345

**Published:** 2026-10-21

**Authors:** Maren Rehders, Helena Kerp, Robert Nica, Rupert Ecker, Dagmar Führer, Klaudia Brix

**Affiliations:** 1 School of Science, Constructor University, Campus Ring 1, 28759 Bremen, Germany; mrehders@constructor.university; 2 Department of Endocrinology, Diabetes and Metabolism, Center Endocrine Medicine (CEM), University Medicine Essen, University of Duisburg-Essen, Hufelandstraße 55, 45147 Essen, Germany; kerp.helena@pediatric-research.org (H.K.); dagmar.fuehrer@uk-essen.de (D.F.); 3 TissueGnostics GmbH, Taborstraße 10/2/8, 1020 Vienna, Austria; robert.nica@tissuegnostics.com (R.N.); rupert.ecker@tissuegnostics.com (R.E.); 4 School of Biomedical Sciences, Faculty of Health, Queensland University of Technology, Brisbane 4059, QLD, Australia; 5 Translational Research Institute, 37 Kent Street, Woolloongabba, Brisbane 4102, QLD, Australia; 6 Faculty of Health Sciences & Medicine, Visiting Academic at Bond University, Gold Coast 4229, QLD, Australia

**Keywords:** cathepsins, extracellular matrix, hypothyroidism, monocarboxylate transporter 8, T4-induced exogenous hyperthyroidism, thyroid gland, thyroid homeostasis, thyroid hormones, thyrocyte proliferation

## Abstract

Adequate thyroid hormone concentrations are essential for tissue homeostasis, including in the thyroid gland. Thyrocyte function is regulated by the hypothalamic–pituitary–thyroid axis and by the coordinated action of thyroglobulin-processing cathepsins and thyroid hormone transporters such as monocarboxylate transporter 8 (Mct8). Here, we investigated the effects of hypothyroidism and thyroxine-induced exogenous hyperthyroidism on thyroid proteases and Mct8 expression in male mice. Thyroid follicular architecture was largely preserved in hyperthyroidism, whereas hypothyroidism resulted in increased thyrocyte extensions, collapsed follicular lumina, and enhanced thyroglobulin turnover. Cathepsin B was reduced in both conditions, pro-cathepsin D declined only in hyperthyroidism, and cathepsin L was upregulated in hypothyroidism. Consistently, thyroglobulin fragments were approximately two-fold more abundant in hypothyroid tissue. Mct8 expression remained unchanged in hyperthyroidism but was nearly absent in hypothyroidism. Hypothyroid tissue also exhibited markedly increased thyrocyte proliferation, while cell death rates were not altered across thyroid dysfunction. In addition, alterations in laminin indicated disbalanced extracellular matrix remodeling in hypothyroidism. Together, these findings demonstrate profound changes in thyroid architecture, proteolytic activity, and thyroid hormone transport under chronic thyroid dysfunction, particularly hypothyroidism. Further studies are needed to define the specific role of individual proteases in these processes.

## 1. Introduction

Thyroid disorders are a common medical concern affecting a large proportion of the world’s population [1,2,3]. Hypothyroidism caused by iodine deficiency can be prevented through dietary iodine supplementation. Congenital hypothyroidism results from defects in thyroid gland development or thyroid hormone (TH) synthesis, whereas acquired hypothyroidism most commonly arises from autoimmune thyroid disease or ablative thyroid treatment, such as radioiodine therapy or surgery. Hyperthyroidism may develop because of autoimmune disorders such as Graves’ Disease (GD) but can also result from thyroid autonomy or thyroiditis [4].

The diagnosis of thyroid dysfunction primarily relies on the measurement of serum thyroid-stimulating hormone (TSH) and, when indicated, circulating TH concentrations. In addition, autoantibodies directed against the TSH receptor and thyroid peroxidase (TPO) are commonly assessed in suspected autoimmune hyperthyroidism and hypothyroidism, respectively [5]. Although diagnostic procedures are highly developed and thyroid dysfunction is generally considered manageable, important challenges remain. For example, many patients report persistent symptoms and reduced well-being, particularly in the context of previous or suspected hypothyroidism, despite having thyroid function parameters within the reference range [1]. Furthermore, the organ-specific consequences of thyroid dysfunction and the effects of hormone replacement therapy (HRT) are often underestimated. Thus, despite the considerable achievements and successes of HRT, the treatment of hypothyroidism continues to present significant clinical challenges. In addition, thyroid status varies across different stages of life, thyroid dysfunction may be transient or persistent, and deviations in circulating TH concentrations from the normal range can affect individual organs to different extents.

We therefore propose that the local TH status of target tissues is of critical importance. This concept likely applies not only to well-established TH target organs such as the brain, heart, kidney, and liver [6] but also to the thyroid gland itself, the sole organ responsible for TH production [7]. To investigate this hypothesis, we analyzed thyroid tissue under physiological (euthyroid) conditions as well as following the induction of hypothyroidism or thyroxine (T4)-induced exogenous hyperthyroidism in male mice using our well-established experimental protocols [8,9].

Experimental investigation of thyroid regulatory mechanisms can be achieved by inducing altered thyroid states or by employing mouse models deficient in proteins essential for thyroid function. For example, defects in TH transport affect the ability of thyrocytes to liberate TH through cathepsin-mediated proteolysis of thyroglobulin (Tg) [10]. Intuitively, one might expect TH liberation from Tg to be reduced when thyrocytes are unable to efficiently export newly liberated T4. Surprisingly, however, the opposite occurs in Mct8-deficient mice: Tg degradation is markedly enhanced, resulting in dysregulated thyroid self-regulation and ultimately leading to thyroid self-thyrotoxicity through the induction of autophagy [10,11].

This present study therefore examined whether Tg-processing cathepsins are affected by T4-induced exogenous hyperthyroidism or by hypothyroidism and how these effects relate to the availability of the major thyroidal TH transporter Mct8. Thyroid tissue architecture was assessed by Cell Profiler™-based phenotyping [12] and tissue image cytometry using our StrataQuest algorithms [13], while Tg-degradation states were investigated using biochemical approaches. In addition, the local microenvironment was analyzed with respect to the extracellular matrix (ECM) component laminin. Our findings indicate that experimentally induced hypothyroidism in mice is associated with enhanced cathepsin L activity, collapsed thyroid follicle lumina, reduced TH export capacity, thyrocyte hyperproliferation and ECM alterations.

## 2. Results

### 2.1. Altered Murine Thyroid Morphology in Experimental Thyroxine-Induced Exogenous Hyperthyroidism and Hypothyroidism

**Figure 1:**
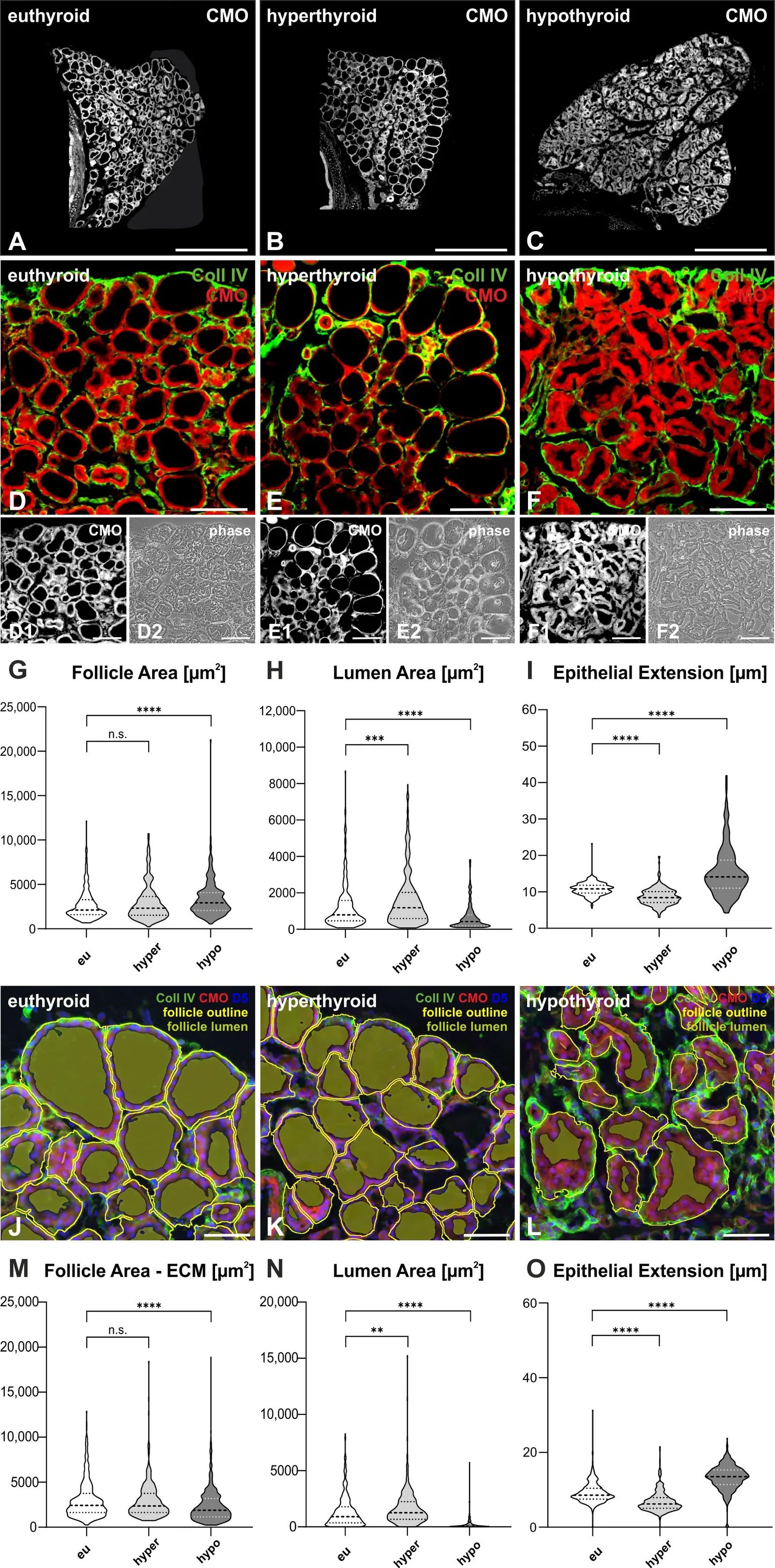
Morphological analysis of euthyroid and hypothyroid mouse thyroid tissue and suppressed thyroid tissue in mice with T4-induced exogenous hyperthyroidism. Middle sections of thyroid lobes from mice in euthyroid (**A**,**D**), T4-induced exogenous hyperthyroid (**B**,**E**), and hypothyroid (**C**,**F**) conditions were stained with CellMaskOrange™ (CMO; grayscale, red; (**A**–**C**,**D**–**F**,**D1**–**F1**)) and immunolabeled with antibodies against collagen IV (Coll IV; green; (**D**–**F**)) as indicated. Merged (**D**–**F**) and single-channel fluorescence images (**A**–**C**,**D1**–**F1**) as well as corresponding phase contrast images (**D2**–**F2**) are depicted as indicated; scale bars represent 500 µm (**A**–**C**) and 100 µm (**D**–**F**). Numbers of animals analyzed: *n* = 2, 2, 3 per thyroid condition. Thyroid follicle morphology was assessed by semi-automated morphometry through a CellProfiler™-based pipeline to determine the follicle areas (**G**), lumen areas (**H**) and epithelial extensions (**I**) in thyroid lobes. The violin plots (**G**–**I**) denote distributions of individual data per analyzed follicles, with mean values indicated as dashed lines ± SD. Numbers of follicles analyzed: *n* = 258 (euthyroid), 283 (T4-induced exogenous hyperthyroid), and 222 (hypothyroid). To confirm the thyroid morphology results obtained with CellProfiler™, the images were additionally viewed with a tissue image cytometry system and analyzed by StrataQuest (**J**–**L**) regarding follicle areas minus ECM (**M**), lumen areas (**N**) and epithelial extensions (**O**) in thyroid lobes. Fluorescence images (**J**–**L**) with outlined follicles and lumen areas are depicted as indicated; scale bars represent 50 µm. The violin plots (**M**–**O**) denote distributions of individual data per analyzed follicle, with mean values indicated as dashed lines ± SD. Numbers of follicles analyzed: *n* = 149 (euthyroid), 154 (T4-induced exogenous hyperthyroid), and 263 (hypothyroid). Levels of significance are indicated as ** for *p* < 0.01, *** for *p* < 0.001 and **** for *p* < 0.0001.

To verify the known effects of chronic thyroid dysfunction on the thyroid gland itself, cryo-sections of thyroid tissue obtained from euthyroid, T4-induced exogenous hyperthyroid and hypothyroid mice were analyzed by semi-automated morphometry using CellProfiler™ (Supplementary Figure S1). Follicle area, lumen area, and epithelial extension (EExt) were thereby determined (Figure 1A–I; Supplementary Figure S2). Moreover, we used our powerful segmentation tools and image analysis in StrataQuest for multi-channel spatial analysis of thyroid tissue phenotypes, thereby confirming the same parameters (Figure 1J–O). As expected, morphological assessment of mid-sections revealed an overall enlargement of the thyroid gland (goiter) in hypothyroid animals, likely resulting from chronic thyrocyte stimulation by elevated blood TSH levels. In addition, thyroid tissue from hypothyroid mice exhibited significantly enlarged follicle areas by 25% (Figure 1G), increased EExt by approx. 43% (Figure 1I), and collapsed colloid, reflected by significantly reduced lumen areas by approx. 50% (Figure 1H), compared with euthyroid control tissue. The follicle areas in thyroid tissue from mice with T4-induced exogenous hyperthyroidism were comparable to those of euthyroid mice. However, in contrast to hypothyroid animals, thyroid follicles of T4-induced exogenous hyperthyroid mice exhibited significantly increased lumen areas by approx. 32% (Figure 1H) and significantly decreased EExt by approx. 20% (Figure 1I) compared with tissue from euthyroid mice.

Tissue segmentation and image analysis by image cytometry yielded comparable results for lumen areas and epithelial extensions, whereas follicle areas minus ECM were significantly decreased by approx. 20% in hypothyroid animals compared with euthyroid controls. This is explained by the fact that, in the StrataQuest analyses, segmentation was by propagation from the ECM marker toward the CMO-labeled epithelium, i.e., excluding the ECM, whereas the CellProfiler™-based analyses assumed that the collagen IV-positive ECM was part of the follicles. Although this represents an approximation, we note that the ECM contribution is considerably higher in hypothyroid than in euthyroid tissue (see below).

Overall, changes in EExt are indicative of an altered activation state of thyrocytes [14] which may be associated with altered Tg synthesis and/or degradation. Therefore, we next performed biochemical analyses of Tg status in thyroid tissue from euthyroid, T4-induced exogenous hyperthyroid, and hypothyroid mice.

### 2.2. Altered Thyroglobulin Degradation in Hypothyroid Mouse Thyroids

**Figure 2:**
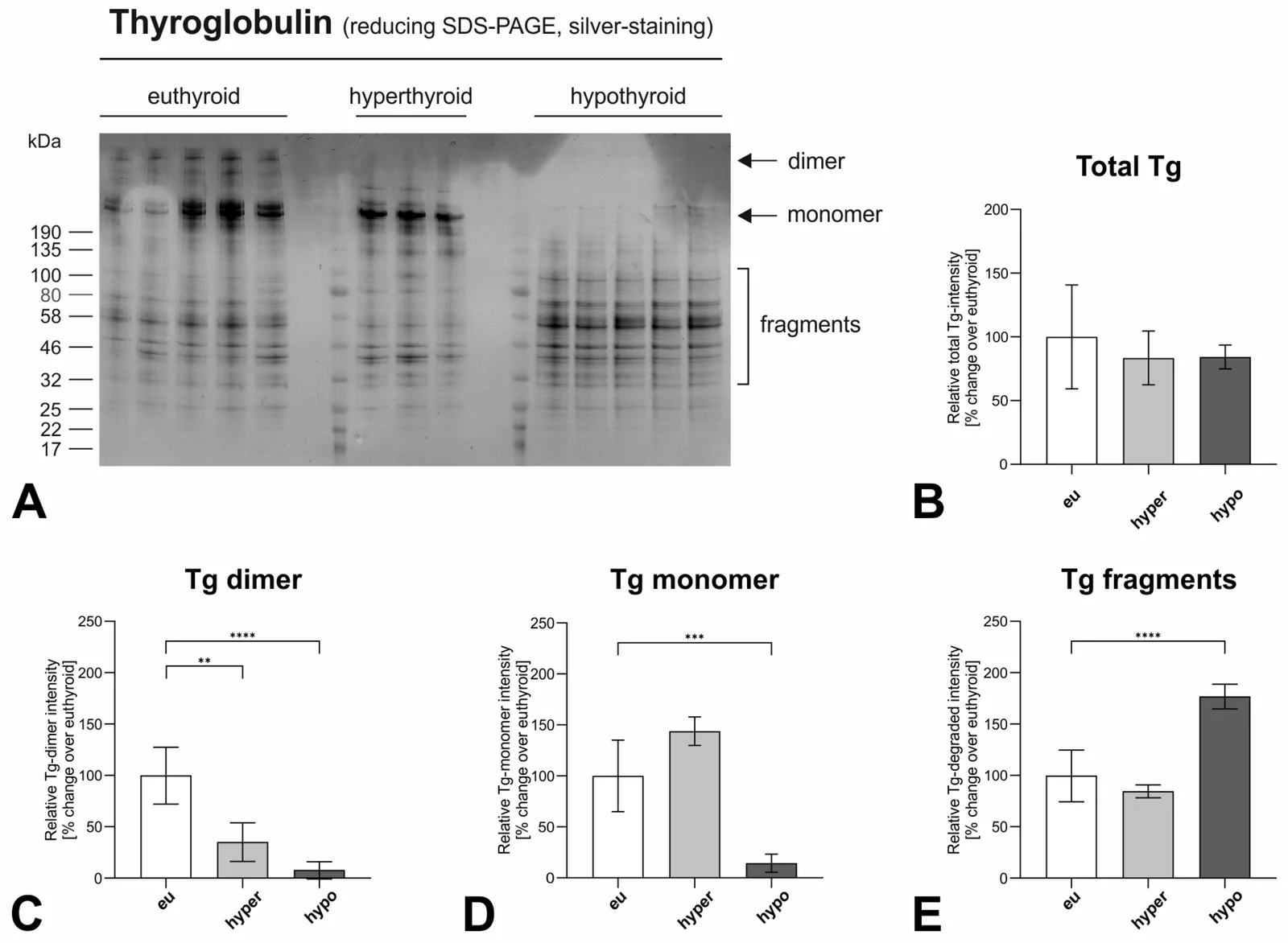
Thyroglobulin status of euthyroid, T4-induced exogenous hyperthyroid, and hypothyroid mice. Proteins of whole thyroid tissue lysates prepared from animals of the indicated thyroid conditions (euthyroid, T4-induced exogenous hyperthyroid, and hypothyroidism) were separated on 8–18% horizontal SDS gels under reducing conditions and silver-stained (**A**). The molecular mass markers are indicated in the left margin. Bands representing Tg dimers, monomers, and fragments are indicated in the right margin. Bar charts represent densitometry analyses of total Tg (**B**), Tg dimers (**C**), Tg monomers (**D**), and Tg fragments (**E**) as % changes over euthyroid controls set to 100%. Animals analyzed: *n* = 5, 3, 5 per condition. Densitometry data was normalized to total silver-stained protein per lane. Bar charts denote the means ± SD. Levels of significance are indicated as ** for *p* < 0.01, *** for *p* < 0.001 and **** for *p* < 0.0001.

To compare Tg oligomerization and degradation across different thyroid conditions with those of euthyroid control mice, thyroid tissue lysates were separated by reducing SDS-PAGE, i.e., under conditions that break disulfide bonds in Tg multimers. This allowed analysis of Tg in its dimeric and monomeric forms, as well as determination of the extent of its processing into smaller fragments by densitometry (Figure 2; Supplementary Figure S3). Total Tg protein levels were comparable across all conditions; however, dimeric Tg was significantly reduced in hypothyroid mice by approximately 92% compared with euthyroid controls, while T4-induced exogenous hyperthyroidism resulted in a reduction of approximately 65%. These results indicate that Tg dimerization is impaired in thyroid dysfunction. Thyroid lysates from T4-induced exogenous hyperthyroid animals contained amounts of Tg monomers and fragments comparable to those observed in euthyroid animals. By contrast, densitometric analysis of bands corresponding to Tg monomers in hypothyroid tissue lysates revealed a significant reduction of approximately 86%. Consequently, the extent of Tg degradation was significantly increased by approximately 77% compared with euthyroid controls. The reduction in dimeric and monomeric Tg forms, together with the corresponding increase in Tg fragments, supports the notion that Tg solubilization and degradation are altered in mice with induced hypothyroidism. By contrast, T4 treatment for thyroid suppression affected Tg cross-linking and/or solubilization but did not affect its degradation.

### 2.3. Dysregulated Expression of Thyroglobulin-Processing Cathepsins in Thyroid Dysfunction

Tg degradation and TH liberation are mediated by the combined action of cathepsins. Therefore, we investigated whether the different extents of Tg proteolysis observed in hypothyroid and T4-induced exogenous hyperthyroid mice could be explained by altered expression levels or maturation states of Tg-processing proteases.

**Figure 3:**
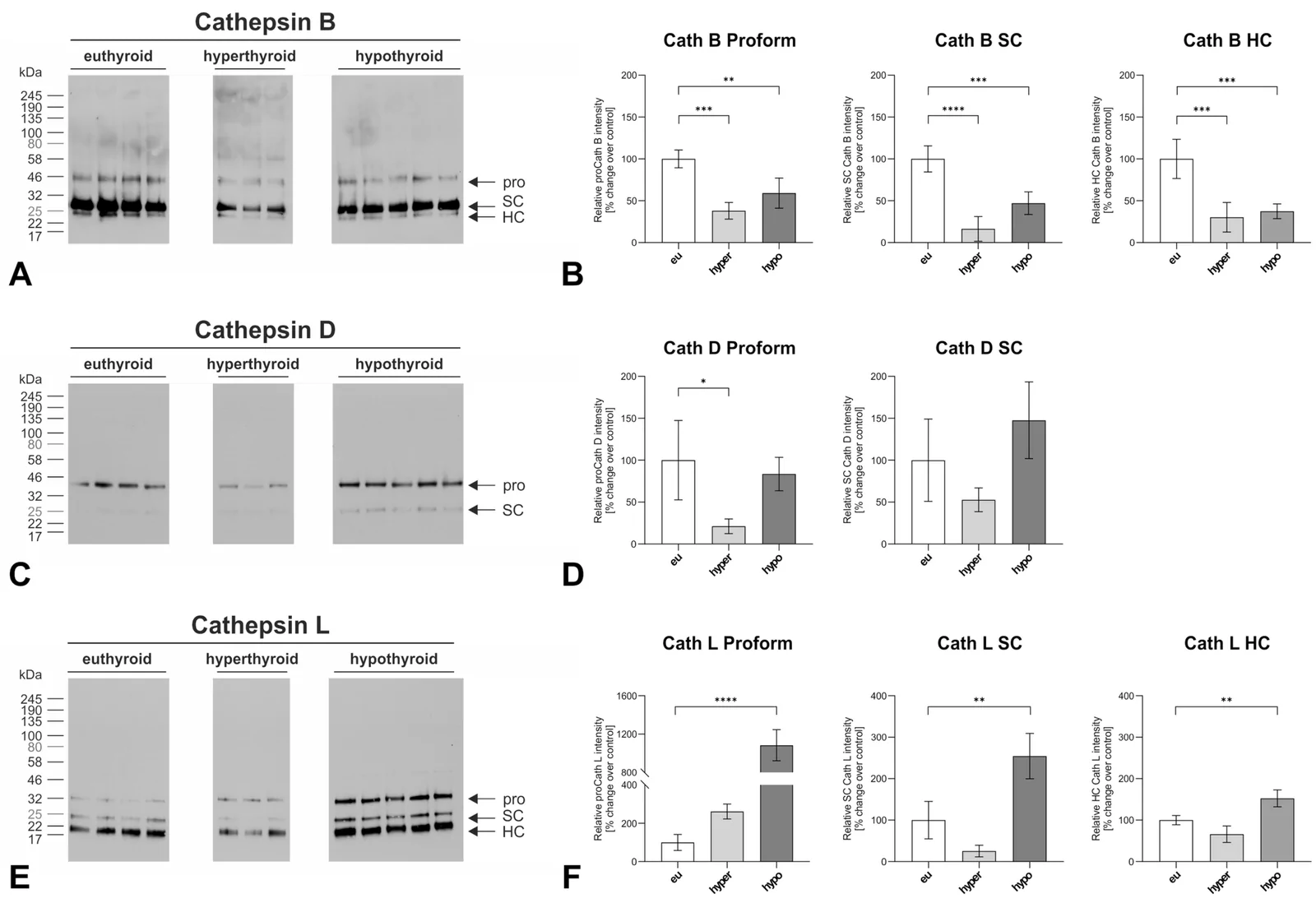
Maturation states of cathepsins B, D, and L are altered in thyroid tissue of T4-induced exogenous hyperthyroid, and hypothyroid mice. Whole thyroid tissue lysates from euthyroid, hypothyroid and T4-induced exogenous hyperthyroid mice were immunoblotted using antibodies specific for cathepsins B, D, or L as indicated ((**A**,**C**,**E**), respectively). Molecular mass markers are indicated in the left margins. Bands representing pro-forms (pro), single-chain (SC), and heavy chain (HC) of two-chain cathepsin forms are indicated in the right margins of the immunoblots. The band densities were normalized to total Ponceau-stained protein per lane. Bar graphs represent amounts of the respective cathepsin forms given as % changes over euthyroid controls set to 100% (**B**,**D**,**F**). Numbers of animals analyzed: *n* = 4, 3, 5 per thyroid condition. Bar charts denote the means ± SD. Levels of significance are indicated as * for *p* < 0.05, ** for *p* < 0.01, *** for *p* < 0.001 and **** for *p* < 0.0001.

Thyroid tissue lysates from euthyroid, T4-induced exogenous hyperthyroid, and hypothyroid animals were separated on horizontal gradient SDS gels, transferred to nitrocellulose membranes, and incubated with antibodies specific for cathepsins B, D, and L to assess the abundance of the different cathepsin forms (Figure 3). Notably, thyroidal cathepsins were detected in their expected pro- and mature forms under all experimental conditions. However, densitometric analysis revealed a significant downregulation of all cathepsin B forms in both T4-induced exogenous hyperthyroid and hypothyroid mice compared with euthyroid controls (Figure 3B). Specifically, thyroidal pro-cathepsin B was reduced by 62% and 41% in T4-induced exogenous hyperthyroidism and hypothyroidism, respectively, whereas the mature single-chain (SC) form and the heavy chain (HC) of the mature two-chain form of cathepsin B were reduced by 84% and 70% in T4-induced exogenous hyperthyroidism and by 53% and 63% in hypothyroidism, respectively, compared with euthyroid controls. Similarly, in T4-induced exogenous hyperthyroid animals, thyroidal pro-cathepsin D levels were reduced by approximately 79%, whereas hypothyroidism did not affect cathepsin D levels relative to euthyroid controls (Figure 3D). Notably, thyroid tissue lysates from mice with T4-induced exogenous hyperthyroidism exhibited no changes in cathepsin L levels compared with euthyroid controls. Hypothyroid animals showed significant increases in both pro- and mature cathepsin L forms, including an approximately 10-fold increase in pro-cathepsin L and 2.5-fold and 1.5-fold increases in the SC form and HC of the two-chain mature form, respectively, compared with thyroid tissue from euthyroid animals (Figure 3F).

### 2.4. Hypothyroidism Induces Apical Re-Localization of Cathepsin L

**Figure 4:**
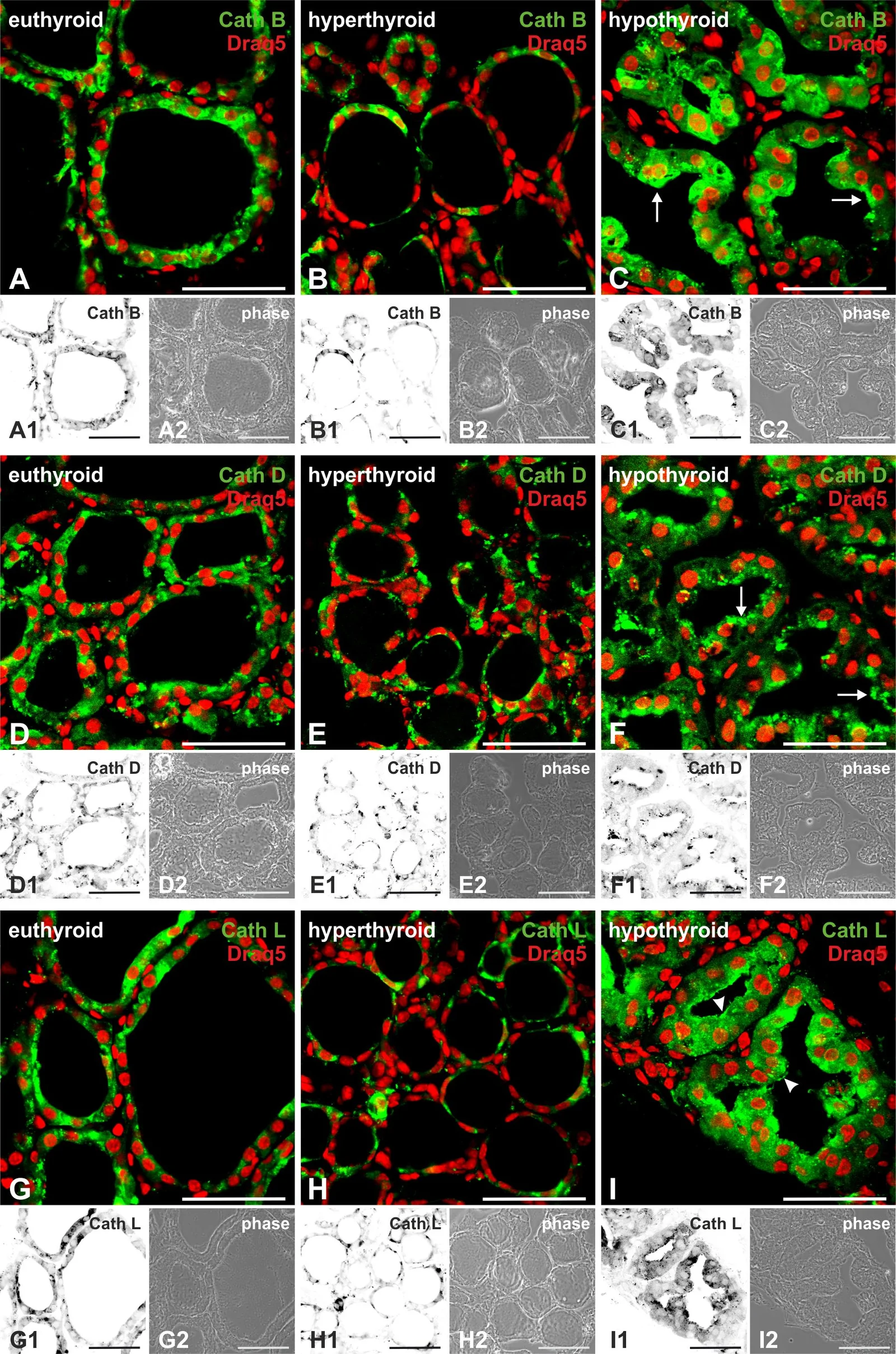
Hypothyroidism results in altered cathepsin localization in mouse thyroid epithelial cells. Cryo-sections of thyroid tissue obtained from euthyroid, T4-induced exogenous hyperthyroid, and hypothyroid mice were labeled with antibodies specific for cathepsin B (green signals in (**A**–**C**)), cathepsn D (green signals in (**D**–**F**)) and cathepsin L (green signals in (**G**–**I**)), while Draq5™ was used as nuclear counterstain (red signals). Merged ((**A**–**C**); (**D**–**F**); (**G**–**I**)) and single-channel fluorescence images of anti-cathepsin-stained tissue (**A1**–**C1**,**D1**–**F1**,**G1**–**I1**) as well as corresponding phase contrast images (**A2**–**C2**,**D2**–**F2**,**G2**–**I2**) are depicted as indicated; scale bars represent 50 µm. In thyroid dysfunction, the cathepsin-positive vesicles, presumably endo-lysosomes, appear more prominently at the apical pole of thyroid epithelial cells rather than in the perinuclear region as in euthyroid controls. Cathepsins B and D maintained a vesicular localization in hypothyroid and T4-induced exogenous hyperthyroid tissue comparable to euthyroid controls ((**B**,**C**), cf. (**A**); (**E**,**F**), cf. (**D**); arrows), while cathepsin L was additionally more prominently localized to the apical plasma membrane of thyrocytes in hypothyroidism ((**I**), cf. (**G**) arrowheads). Representative images for cathepsins B and D were obtained from thyroid tissue of three euthyroid and hypothyroid and two T4-induced exogenous hyperthyroid animals, and representative images for cathepsin L were obtained from thyroid tissue of three animals per condition.

To investigate whether cathepsins are differentially localized in thyroid tissue under altered thyroid states, cryo-sections of thyroid glands from euthyroid, hypothyroid, and T4-induced exogenous hyperthyroid mice were labeled with antibodies against cathepsins B, D, and L and examined by confocal laser scanning microscopy (Figure 4; Supplementary Figures S4–S6). Cathepsins B and D were localized to the endo-lysosomal compartments of thyroid epithelial cells under euthyroid conditions, and their sub-cellular localization remained unchanged in both T4-induced exogenous hyperthyroid and hypothyroid mice. By contrast, cathepsin L immunofluorescence signals were increased in hypothyroid tissue, as expected from the marked upregulation of its pro-form (see above), and were additionally detected at the apical plasma membrane of thyroid epithelial cells (Figure 4I, arrowheads). By comparison, its vesicular localization was maintained in T4-induced exogenous hyperthyroidism.

### 2.5. Hypothyroidism Is Associated with Reduced Mct8 Protein Levels

**Figure 5:**
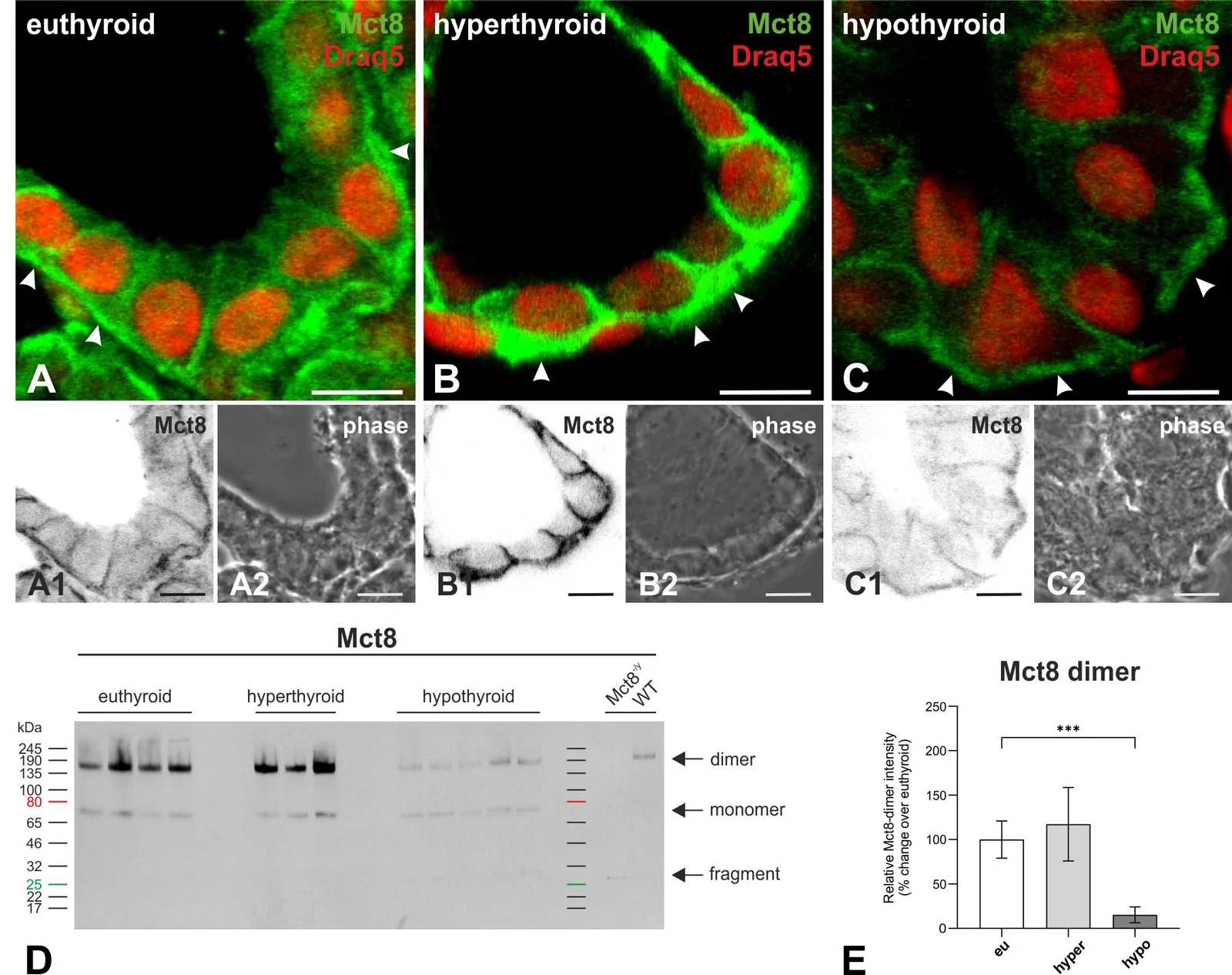
Hypothyroidism results in downregulation of Mct8 in mouse thyroid tissue. Cryo-sections of thyroid tissue obtained from euthyroid, T4-induced exogenous hyperthyroid, and hypothyroid mice were labeled with antibodies specific for Mct8 (green signals in (**A**–**C**)), while Draq5™ was used as nuclear counterstain (red signals). Merged (**A**–**C**) and single-channel fluorescence images of stained tissue (**A1**–**C1**) as well as corresponding phase contrast images (**A2**–**C2**) are depicted as indicated; scale bars represent 10 µm. Mct8 maintained its basolateral localization in thyroid epithelial cells of hypothyroid and T4-induced exogenous hyperthyroid tissue, i.e., comparable to euthyroid controls (arrowheads). Thyroid tissue lysates from euthyroid, hypothyroid and T4-induced exogenous hyperthyroid mice were immunoblotted using an antibody specific to Mct8 (**D**). Molecular mass markers are indicated in the left margin. Bands representing dimeric and monomeric forms of Mct8 as well as a small molecular mass fragment are indicated in the right margin of the immunoblot. The band densities were normalized to total Ponceau-stained protein per lane. Bar graphs represent amounts of dimeric Mct8 given as % changes over euthyroid controls set to 100% (**E**). Numbers of animals analyzed: *n* = 4, 3, 5 per thyroid condition. Bar charts denote the means ± SD. Levels of significance are indicated as *** for *p* < 0.001.

The major TH transporter Mct8 mediates T4 export from and import into thyroid epithelial cells and thus contributes to the determination of thyroid status not only in peripheral tissues but also within the thyroid gland itself. Because the increased abundance of cathepsin L and its relocation to the follicular lumen and apical plasma membrane of thyroid epithelial cells in hypothyroidism (see above) were reminiscent of our previous observations in Mct8-deficient mice [10], we focused on the expression and localization of this TH transporter in thyroid tissue from mice with T4-induced exogenous hyperthyroidism and hypothyroidism (Figure 5; Supplementary Figure S7).

Mct8 was detectable at the basolateral plasma membrane domains of thyroid epithelial cells in tissue from euthyroid, hypothyroid, and T4-induced exogenous hyperthyroid mice alike (Figure 5A–C). However, when thyroid tissue lysates of euthyroid, hypothyroid, and T4-induced exogenous hyperthyroid animals were separated on horizontal gradient SDS gels and immunoblotted with antibodies specific for Mct8 (Figure 5D), dimeric and monomeric forms of Mct8 were detected under euthyroid and T4-induced exogenous hyperthyroid conditions, whereas Mct8-immunoreactive bands were almost undetectable in hypothyroid animals. Remarkably, densitometric analysis of Mct8-immunopositive bands (Figure 5E) revealed a significant downregulation in the prominent dimeric form by approximately 85% in hypothyroid animals compared with euthyroid controls. By contrast, Mct8 protein levels remained unchanged in thyroid tissue lysates from T4-induced exogenous hyperthyroid mice.

### 2.6. Enhanced Thyrocyte Proliferation in Hypothyroid Mice

**Figure 6:**
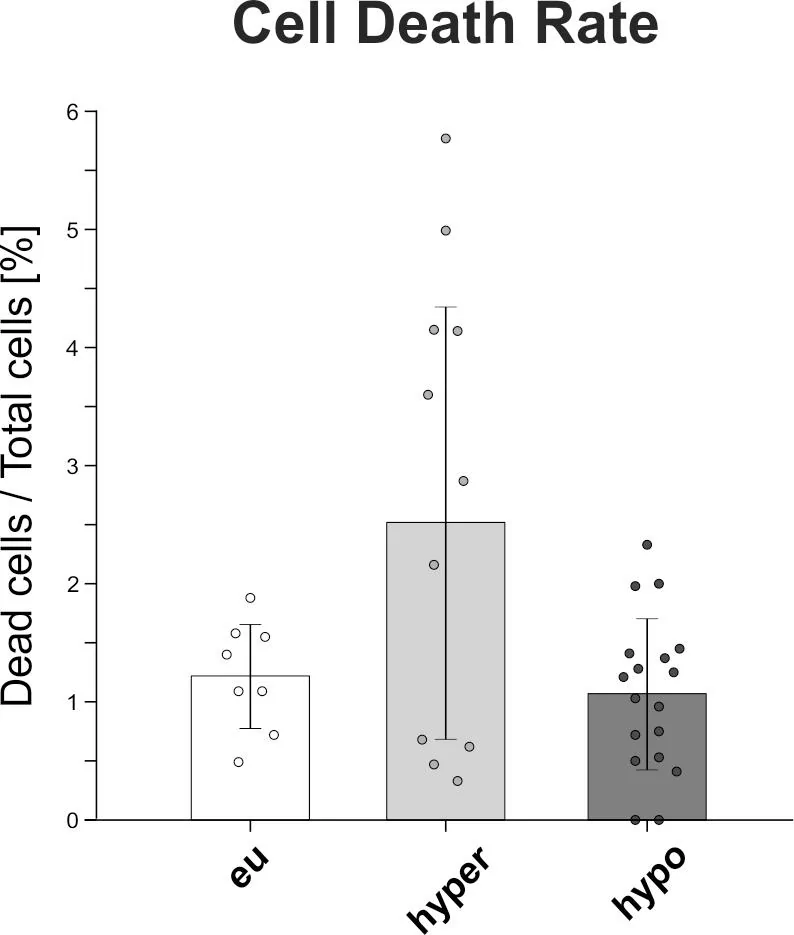
Cell death rates in euthyroid, hyperthyroid, and hypothyroid conditions. Cryo-sections from thyroid tissue obtained from euthyroid, hypothyroid and T4-induced exogenous hyperthyroid mice were labeled with Draq5™ as nuclear stain. Representative images were obtained, and CellProfiler™ was used to count total cell numbers, while manual inspection yielded the numbers of dead cell remnants within thyroid follicular lumina. Bar charts denote the means ± SD, circles indicate cell death rate per analyzed image. Numbers of animals analyzed: *n* = 2, 2, 3 per thyroid condition. Numbers of images analyzed: *n* = 8 (eu), *n* = 11 (hyper), *n* = 18 (hypo).

The downregulation of Mct8 suggested diminished TH export and/or import by thyrocytes in hypothyroid mice, which could lead to intracellular TH accumulation. In combination with the enhanced Tg degradation observed under these conditions, this may ultimately result in self-thyrotoxic effects. Therefore, we analyzed cell death rates in thyroid tissue from mice representing different thyroid states (Figure 6). Dead cell remnants within thyroid follicle lumina were quantified, which revealed 1.22% ± 0.44 dead cells relative to the total number of cells under euthyroid control conditions (*n* = 259 follicles). By contrast, T4-induced exogenous hyperthyroidism resulted in a cell death rate of 2.52% ± 1.83 (*n* = 283 follicles), which did not differ significantly from that of euthyroid follicles and was also not different from the cell death rate of 1.05% ± 0.65 observed in hypothyroid follicles (*n* = 224). Death rates were approximately normally distributed in all groups (Shapiro–Wilk tests, all *p* > 0.05). However, variances differed significantly among groups (Levene’s test: F(2,34) = 5.82, *p* = 0.0067). Consequently, Welch’s ANOVA was used and showed no significant effect of group on death rate, F(2,20.55) = 2.02, *p* = 0.158. Thus, no statistically significant differences in mean death rates were detected among the control, hyperthyroid, and hypothyroid groups (Figure 6).

These findings indicate that neither T4-induced exogenous hyperthyroidism nor the reduction in Mct8-mediated TH export by hypothyroid thyrocytes upon the induction of hypothyroidism by treatment with a low iodine diet, methimazole (MMI), and sodium perchlorate (LoI/MMI/ClO_4_^−^] led to increased thyrocyte death.

**Figure 7:**
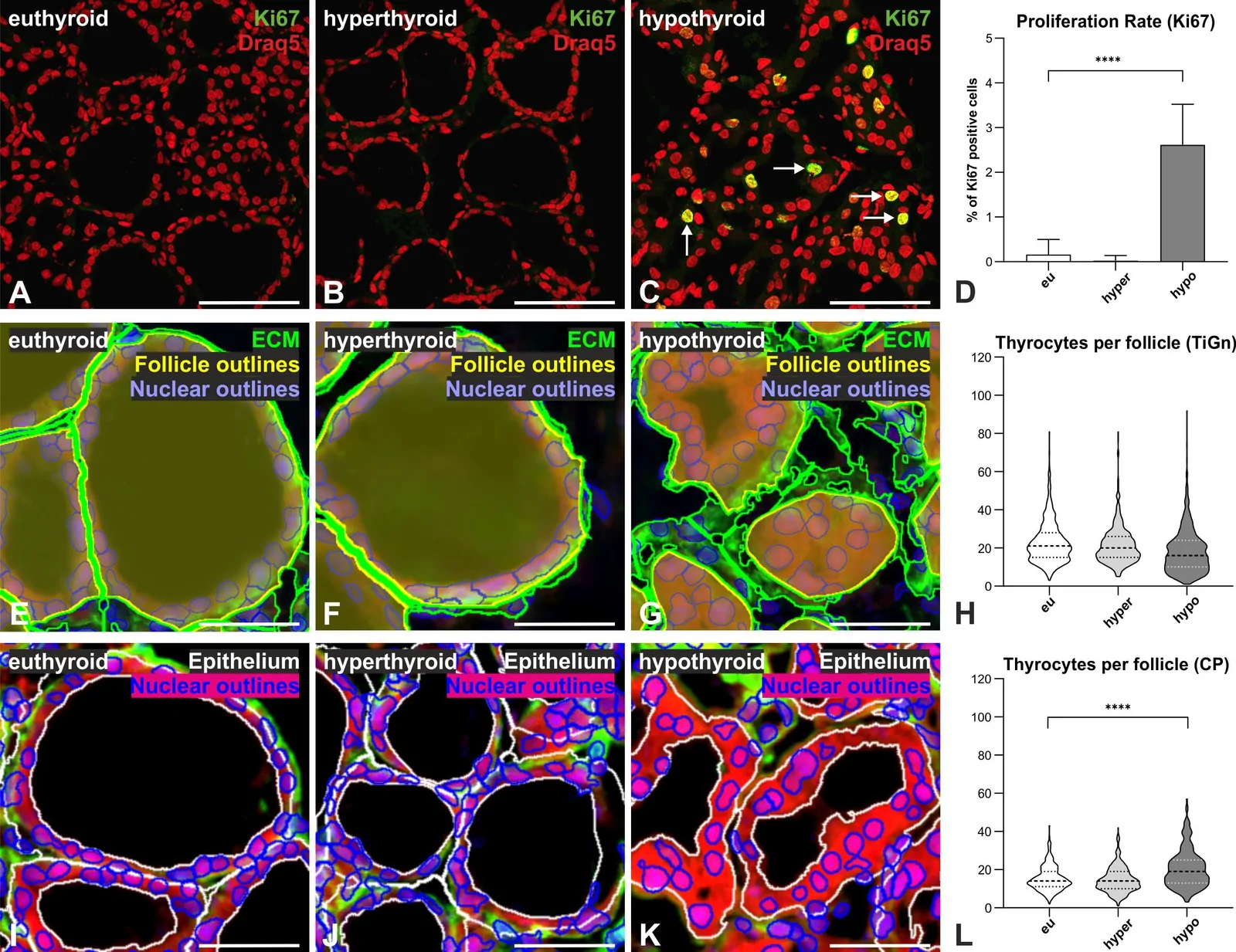
Hypothyroidism results in hyperproliferation of mouse thyroid epithelial cells. Cryo-sections from thyroid tissue obtained from euthyroid, hypothyroid and T4-induced exogenous hyperthyroid mice were labeled with antibodies specific for the proliferation marker Ki67 (green signals in (**A**–**C**)), while Draq5™ was used as nuclear counterstain (red signals in (**A**–**C**)). Merged (**A**–**C**) fluorescence micrographs of stained tissue are depicted as indicated; scale bars represent 50 µm. High numbers of Ki67-positive cells were detected in thyroid tissue of hypothyroid animals ((**C**), arrows), whereas only very few Ki67-positive cells were detected in euthyroid and T4-induced exogenous hyperthyroid tissue ((**A**) and (**B**), respectively). Representative images were obtained from three animals per condition. CellProfiler™ was used to count Ki67-positive cells compared to total cell numbers, yielding the proliferation rates (**D**). Numbers of thyroid cells analyzed: *n* = 5253 (eu), *n* = 4420 (hyper), *n* = 4701 (hypo). Bar charts denote means + SD. Levels of significance are indicated as **** for *p* < 0.0001. For each thyroid condition, the total cell numbers per follicle were counted by StrataQuest (**E**–**H**) and CellProfiler™ (**I**–**L**). Fluorescence micrographs of stained tissue with follicle and nuclear outlines are depicted as indicated (**E**–**G**,**I**–**K**); scale bars represent 100 µm. Note that mitotic cells are occasionally counted as one nucleus by StrataQuest, which explains the differences compared to CellProfiler™-based enumeration ((**L**), cf. (**H**)).

Given the observed increase in thyroid gland size in mice with induced hypothyroidism (see above), we next investigated whether thyrocyte proliferation rates were altered under different thyroid states. Cryo-sections of thyroid tissue from euthyroid, hypothyroid, and T4-induced exogenous hyperthyroid mice were immunolabeled with antibodies specific for the proliferation marker Ki67 (Figure 7A–D; Supplementary Figure S8). Quantification of proliferating *versus* total cells using CellProfiler™ analysis revealed a 16-fold increase in proliferation rates in thyroid tissue from hypothyroid mice compared with euthyroid controls, whereas T4-induced exogenous hyperthyroidism did not alter proliferation rates.

StrataQuest analyses revealed fewer cells per thyroid follicle (Figure 7E–H) in hypothyroid tissue, whereas CellProfiler™-based cell enumeration showed significantly more cells per follicular epithelium (Figure 7I–L). This discrepancy can be explained by differences in the segmentation criteria regarding mitotic figures, which were refined through additional manual editing in CellProfiler™-based analyses (Figure 7K). As a result, late mitotic stages were scored as two cells in the CellProfiler™ workflow, whereas StrataQuest segmentation classified comparable mitotic stages as a single cell (Figure 7G). Taken together, these observations indicate that Ki67 labeling provided the most reliable measure of proliferating cells (Figure 7C).

### 2.7. Altered Laminin Distribution and Extracellular Matrix Remodeling in Hypothyroidism

**Figure 8:**
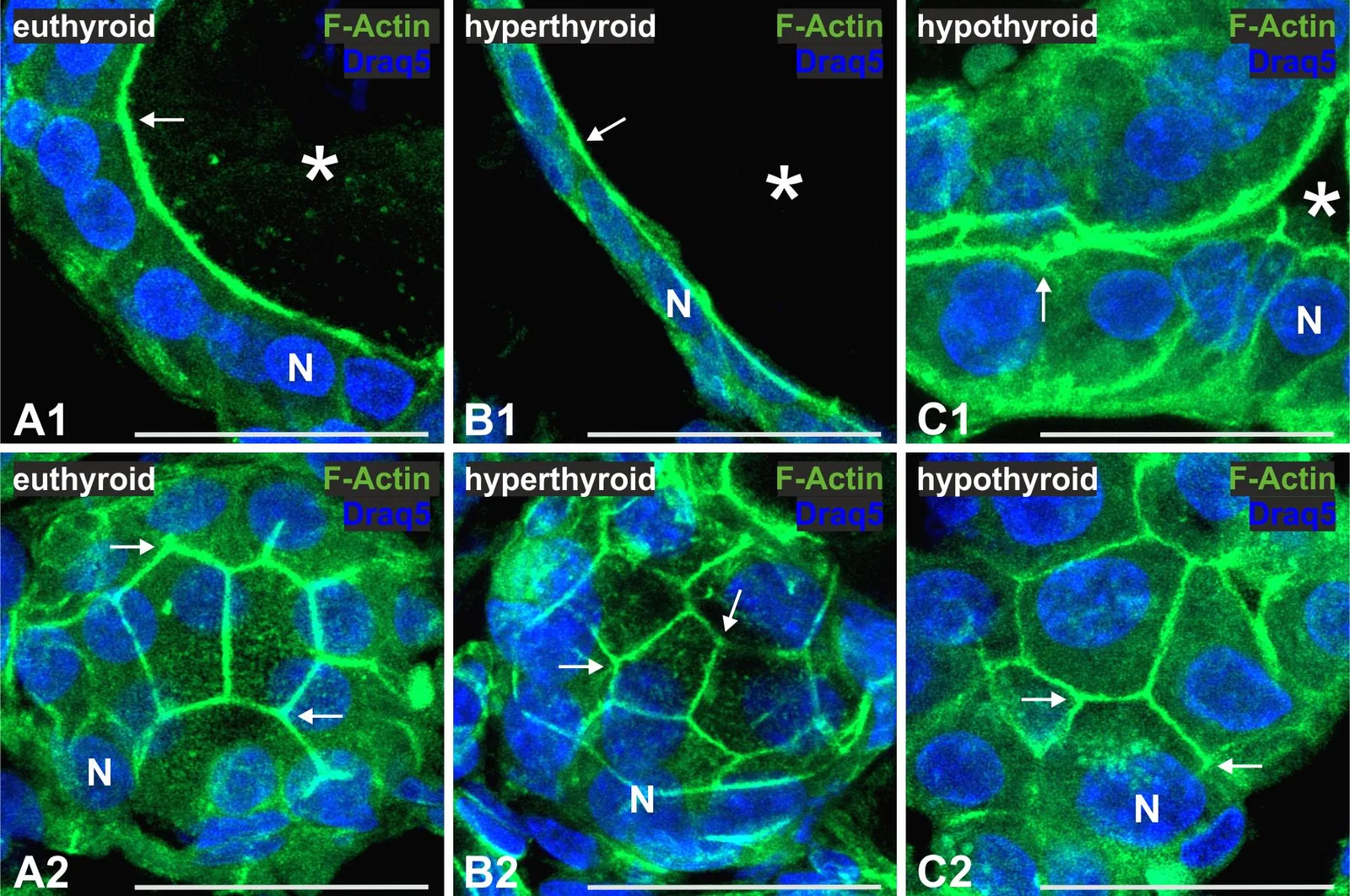
Thyroid epithelial tightness is maintained in mice of different thyroid states. Cryo-sections from thyroid tissue obtained from euthyroid, T4-induced exogenous hyperthyroid, and hypothyroid mice were labeled with phalloidin binding to F-actin, which is particularly rich underneath the apical (**A1**–**C1**) and basolateral (**A2**–**C2**) plasma membrane (green signals), while Draq5™ was used as nuclear counterstain (blue signals). Merged fluorescence micrographs (**A1**–**C2**) of stained tissue are depicted as indicated. N denotes nuclei; scale bars represent 25 µm. The filamentous cortex underneath the apical and basolateral plasma membrane domains appeared continuously and at comparable phalloidin-staining intensity in all thyroid conditions, which indicates tight thyroid epithelia toward the follicle lumina (asterisks in (**A1**–**C1**)) and intact borders between neighboring cells (**A2**–**C2**). Dot-like tight junction-resembling connections (arrows) between neighboring cells in all three models support this conclusion. Representative images were obtained from thyroid tissue of 3 animals per condition. Numbers of images analyzed: *n* = 11 (eu), *n* = 12 (hyper), *n* = 12 (hypo). “*” denotes follicle lumina.

Because thyroid follicles in hypothyroid mice did not exhibit a spherical morphology, we investigated the integrity of the follicle-limiting boundaries. To this end, cryo-sections of thyroid tissue from euthyroid, T4-induced exogenous hyperthyroid, and hypothyroid mice were stained with phalloidin to analyze the F-actin cytoskeleton underlying the apical and basolateral plasma membrane domains of thyroid epithelial cells (Figure 8; Supplementary Figure S9). Notably, no alterations in the cortical microfilament network were observed under either condition. Both hypothyroid and T4-induced exogenous hyperthyroid tissues exhibited F-actin staining at the apical and basolateral poles of thyrocytes. These observations indicate that the structural integrity of the thyroid epithelium facing the thyroid follicular lumen was maintained, thereby further excluding cell stress or general toxicity of the experimental treatments used to induce the different thyroid states.

**Figure 9:**
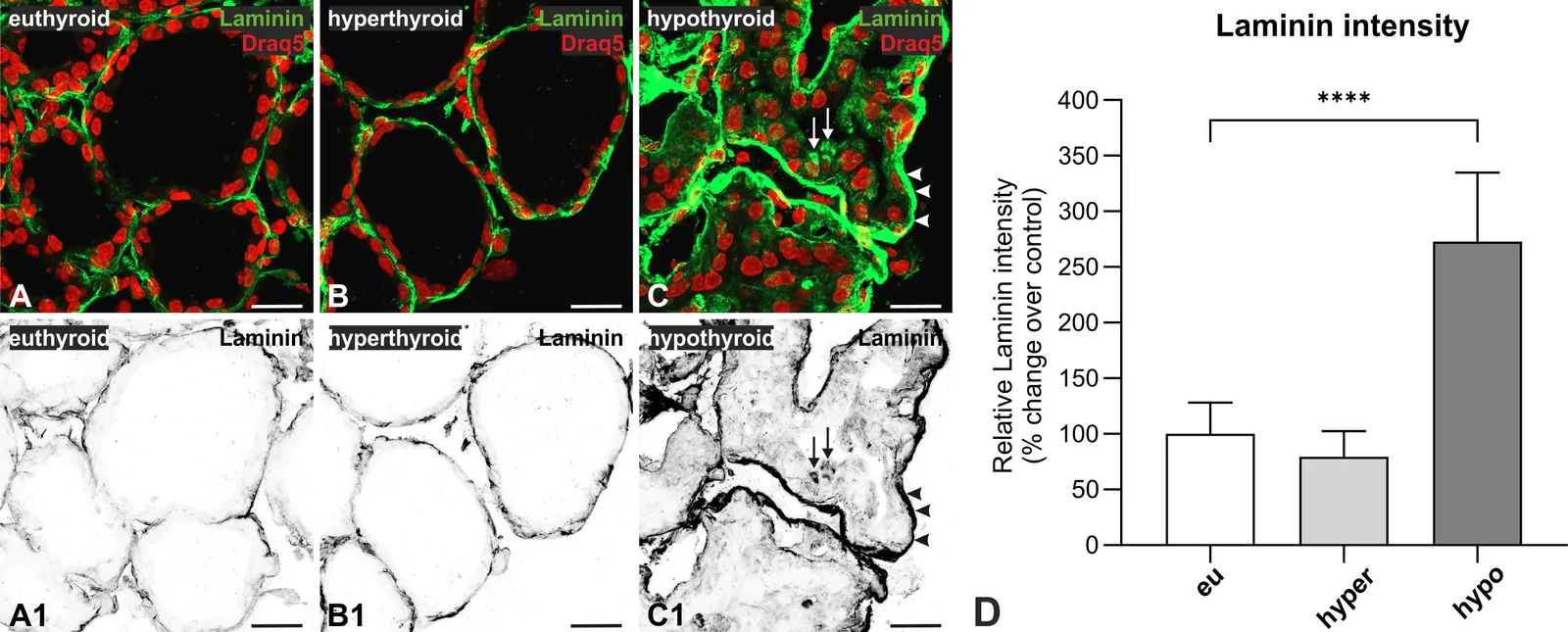
Hypothyroidism results in increased laminin signals and intracellular localization in mouse thyroid epithelial cells. Cryo-sections from thyroid tissue obtained from euthyroid, T4-induced exogenous hyperthyroid, and hypothyroid mice were labeled with antibodies specific for laminin (green signals (**A**–**C**), grey signals in (**A1**–**C1**)), while Draq5™ was used as nuclear counterstain (red signals in (**A**–**C**)). Merged (**A**–**C**) and single-channel fluorescence micrographs (**A1**–**C1**) of stained tissue are depicted as indicated; scale bars represent 50 µm. Laminin maintained its basal localization in the extracellular matrix surrounding individual follicles in all thyroid conditions, which was increased in T4-induced exogenous hyperthyroidism (arrowheads in (**C**,**C1**)) and additionally found in intracellular vesicles (arrows). Representative images were obtained from thyroid tissue of 3 animals per condition. CellProfiler™ was used to determine the mean image intensity of the laminin signal and to count the number of Draq5™-stained nuclei per image. The graph displays the mean laminin intensity of eu-, T4-induced exogenous hyperthyroid, and hypothyroid animals, normalized to the total number of Draq5™-stained nuclei per image (**D**). Numbers of animals analyzed *n* = 3 per condition; number of images analyzed: *n* = 9 (eu), *n* = 8 (hyper), *n* = 9 (hypo). Bar charts denote means + SD. Levels of significance are indicated as **** for *p* < 0.0001.

Because the thyroid gland is rich in connective tissue, cryo-sections of thyroid tissue were additionally immunolabeled with antibodies specific for laminin (Figure 9; Supplementary Figure S10). As expected, this ECM component was detected in the basal lamina surrounding thyroid follicles in euthyroid tissue, and its localization was not altered by T4-induced exogenous hyperthyroidism. By contrast, induced hypothyroidism in mice resulted in markedly increased laminin immunofluorescence signals compared with euthyroid controls. Moreover, laminin levels were increased approximately 2.8-fold and were detected not only in the basal lamina surrounding non-spherical, distorted thyroid follicles but also intracellularly within vesicular structures of thyrocytes under hypothyroid conditions. These observations indicate altered ECM deposition and/or remodeling in hypothyroidism, whereas the basal lamina of thyroid follicles from T4-induced exogenous hyperthyroid animals appeared unaffected.

### 2.8. Summary of Results

The main findings of this study are summarized in Table 1, which provides an overview of the biochemical and morphological analyses.

## 3. Discussion

### 3.1. Thyroid Self-Regulation

It is widely accepted that thyrocyte activity, aimed at maintaining physiological TH concentrations, must be self-regulated through mechanisms that are integrated with canonical regulation mediated by the hypothalamic–pituitary–thyroid (HPT) axis. This notion is based on the observation that individual thyroid follicles exhibit remarkable functional autonomy, which is reflected by their morphological heterogeneity [7,10]. In other words, the hundreds of follicles within a thyroid lobe may differ in size, epithelial extension, and luminal content under both physiological and pathological thyroid conditions throughout the lifetime of an animal or human [10,11,14,15,16]. This observation is particularly intriguing because thyroid follicles and blood vessels are closely associated within so-called angio-follicular units [17]. Consequently, each follicle within a thyroid lobe is, in principle, exposed to the same circulating concentration of TSH, yet individual follicles respond differently to HPT axis-mediated TSH stimulation. It is therefore evident that local mechanisms of thyroid self-regulation must exist to explain the heterogeneity observed among follicles within the same thyroid lobe.

The present study builds upon the concept that the self-regulation of thyroid epithelial cells is not yet fully understood and involves several non-canonical mechanisms in addition to canonical HPT axis regulation [7]. These include coregulation of thyrocytes through G protein-coupled receptors at the apical and basolateral plasma membrane domains, namely, trace amine-associated receptor 1 (Taar1; non-canonical) and the TSH receptor (canonical), respectively. Furthermore, transmembrane TH transporters at different locations contribute to thyroid self-regulation by controlling TH flux across apical and basolateral plasma membranes as well as from endo-lysosomal compartments, where most TH liberation occurs [10,11,18,19].

In addition, Tg itself has emerged as an important regulatory factor. High concentrations of Tg stored within the follicular lumen as compacted and covalently cross-linked colloid inhibit Tg solubilization and degradation, thereby restricting TH utilization by activated thyrocytes [20,21]. Likewise, elevated circulating iodide concentrations maintain thyroid follicles in a resting state [22], which indicates that iodinated Tg is not only the principal precursor of TH but also an important component of thyroid self-regulation.

Another regulatory level is established through the finely balanced interplay of proteolytic enzymes and their endogenous inhibitors (reviewed in [23]). Together, these factors govern the storage and processing states of the macromolecular TH precursor Tg within the follicular lumen [7,14,23]. In particular, the aspartyl protease cathepsin D and the cysteine proteases cathepsins B and L represent major Tg-processing enzymes that mediate TH liberation from Tg at distinct peri- and intracellular locations [7,23,24,25]. In addition to these ubiquitously expressed cathepsins, cathepsins C, K, S, and V/L2, as well as plasma glutamate carboxypeptidase and plasminogen-like protease, contribute to Tg processing [7,23,26,27]. Although the cleavage sites of the major Tg-processing enzymes are mapped onto the Tg structure in elegant studies [28,29], the regulation of protease expression by TSH remains poorly understood. Cathepsins B, D, and S have been reported to be upregulated during chronic TSH stimulation, such as occurs in hypothyroidism. However, only a limited number of studies have addressed this topic experimentally using rabbit [26], rat [30,31], or human thyroid epithelial cells [32].

### 3.2. Interplay of Thyroglobulin-Processing Cathepsins and Mct8 with Thyroid Structural Alterations in Hypothyroidism

Previously, we observed an interdependence between TH transporters and cysteine cathepsin-mediated Tg processing using corresponding mouse models [10,11]. Mechanistically, deficiency in either TH transporter Mct8 or L-type amino acid transporter 2 (Lat2) resulted in autophagy induction, which explains the increased abundance of cathepsins and enhanced Tg processing that ultimately led to self-thyrotoxicity [11]. Autophagy is unlikely to account for the observations reported here because the various cathepsins responded differently in hypothyroid and T4-induced exogenous hyperthyroid conditions. Thus, our findings point to a cathepsin L-specific effect rather than a global upregulation of endo-lysosomal enzymes.

Given the previously observed and somewhat counterintuitive interplay between TH transporters and Tg-processing cathepsins, we examined whether intra-thyroidal thyroid states were associated with alterations in Mct8. Accordingly, we assessed Mct8 expression and localization in thyroid tissue from mice with hypothyroidism and T4-induced exogenous hyperthyroidism, conditions characterized by altered TH levels as well as changes in cathepsin B, D and L abundance and maturation. We focused on Mct8 because it represents the major TH transporter in murine thyrocytes, whereas Mct10, Lat1, and Lat2 are expressed at substantially lower levels. Importantly, no compensatory regulation among TH transporters has been observed in TH transporter-deficient mice [19]. Mct8 was detected in both dimeric and monomeric forms (see Figure 5). Because Mct8 predominantly exists as a stable homodimer [33,34], we focused our analyses on the dimeric form, which was reduced by approximately 85% under hypothyroid conditions, whereas no significant changes were detected in T4-induced exogenous hyperthyroidism. This pronounced reduction in Mct8 suggests impaired TH transport by hypothyroid thyrocytes, which potentially results in intracellular TH accumulation and subsequent cytotoxic effects. However, neither self-thyrotoxicity nor toxicity resulting from LoI/MMI/ClO_4_^−^ treatment is supported by our data (see Figure 6), as the number of dead cell remnants within hypothyroid follicular lumina did not differ from that observed under euthyroid conditions.

Interestingly, the near absence of Mct8 under hypothyroid conditions resembled our previous observation of markedly reduced Mct8 expression in thyroid carcinoma [35]. In that study, Mct8 expression differed significantly between benign and malignant thyroid lesions, with reduced Mct8 levels being associated with malignancy [35]. We therefore investigated whether experimentally induced thyroid dysfunction was accompanied by alterations suggestive of neoplastic transformation. Indeed, hypothyroid thyroid tissue displayed markedly increased proliferative activity. The observed increase in proliferation was unexpected, particularly because hypothyroidism is characterized by elevated circulating TSH levels together with a pronounced reduction in Mct8. By contrast, our earlier in vitro studies using the rat thyroid epithelial cell line PCCl3 demonstrated a TSH-induced upregulation of Mct8, an effect that was also observed in thyroid tissue from patients with GD [35]. The latter observation may indicate an additional contribution of immune-mediated mechanisms in patients, which were absent in both the PCCl3 cell culture model and the present study.

We further investigated whether thyroid dysfunction affects ECM deposition and remodeling. Remarkably, laminin levels were substantially increased in thyroid tissue from hypothyroid mice (see Figure 9). These findings suggest a second feature commonly associated with premalignant tissue remodeling, namely, altered ECM organization. Notably, elevated serum TSH concentrations have been associated with both an increased incidence of thyroid cancer and more advanced tumor stages [36].

Future long-term studies will be required to investigate additional markers of epithelial-to-mesenchymal transition (EMT) and related pathways. Such studies may further substantiate our hypothesis that experimentally induced hypothyroidism leads to Mct8 downregulation while simultaneously enhancing cathepsin L-mediated Tg degradation and TH liberation, thereby promoting tissue alterations that may represent an early premalignant state.

### 3.3. Clinical Context, Perspectives, and Limitations of This Study

The experimental induction of hyperthyroidism or hypothyroidism in mice has been achieved using several protocols, including those employed in the present study, as detailed by us previously [8,9]. Ideally, a larger sample size comprising both female and male mice would have been analyzed. Future studies should include a larger number of animals of both biological sexes, as sex and age are important confounding factors in thyroid dysfunction [37,38].

Because hyperthyroidism in humans is typically associated with autoimmune disorders such as GD, which is characterized by autoantibodies directed against the TSH receptor, long-term murine models of hyperthyroidism have been established through the injection of plasmids encoding the TSH receptor or its subunits to induce immunization [39]. In the present study, however, our primary aim was to analyze the effects of excessive TH-induced hyperthyroidism without contributions from the immune system. Therefore, we employed our well-established protocol for T4-induced exogenous hyperthyroidism [8].

Likewise, hypothyroidism is commonly observed in autoimmune conditions such as Hashimoto’s disease [1]. However, hypothyroidism may also result from thyroid ablation surgery, for example, in patients with suspected thyroid cancer [40]. It has become clear that, although thyroid nodules are frequently detected, only a small proportion is malignant. Therefore, overdiagnosis and overtreatment remain major concerns [40]. Moreover, the elevated serum TSH concentrations characteristic of hypothyroidism are thought to contribute to tissue alterations, as TSH acts as a growth factor in a variety of cell types, including, most notably, thyrocytes. This may promote the progression of pre-existing malignancies. However, clinical evidence linking subclinical hypothyroidism to cancer risk and the progression of pre-existing lesions remains limited [41]. Furthermore, the precise mechanistic links between thyroid dysfunction and various cancers, including thyroid cancer, have yet to be fully elucidated [42,43]. There is growing consensus that the diagnosis of thyroid cancer requires biomarker testing beyond the analysis of genetic mutations alone [44].

In this study, we detected a hyperproliferation of thyrocytes and alterations in ECM composition in thyroid tissue from hypothyroid mice. Although increased cell proliferation, altered ECM deposition, and tissue remodeling are components of the EMT program (see above), these findings cannot be considered definitive evidence of premalignant states. Rather, they should be interpreted with caution, as they may also arise because of the experimental conditions used to induce thyroid dysfunction.

The induction of hypothyroidism by a low-iodine diet combined with MMI and sodium perchlorate treatment or deficiencies in thyroid-specific proteins can be associated with severe toxic effects, as previously reported by several groups [45,46,47,48]. In the present study, thyroid tissue from hypothyroid mice did not exhibit signs of cell death, which excludes the possibility that luminal cellular debris might serve as a source of TH biosynthesis, as suggested by Zhang-X et al. [47], thereby compromising the hypothyroid state. Furthermore, the lack of intact Tg observed in thyroid tissue from hypothyroid mice could be expected to impose substantial stress on thyrocytes, including endoplasmic reticulum (ER) stress or the induction of autophagy, as previously described [49]. Autophagy induction has also been observed in Mct8-deficient mouse models (see above and [11]). Therefore, one might speculate that the markedly reduced abundance of Mct8 detected under hypothyroid conditions in this study could similarly promote autophagy. However, in the hypothyroid model employed here, generated by a low-iodine diet combined with MMI and sodium perchlorate treatment, differential effects on endo-lysosomal enzymes were observed (see Figure 3 and Figure 4), and no evidence of lipidated LC3 associated with newly formed autophagic vesicles was detected.

Additional indicators of cellular stress have been linked to alterations in the actin cytoskeleton and its connections to tight and adherens junctions between thyrocytes. Therefore, we further examined the F-actin cytoskeleton beneath the apical and basolateral plasma membrane domains as a potential marker of structural alterations associated with thyroid dysfunction, as reported previously [50]. However, no obvious disruption of microfilament organization at either the thyrocyte pole or plasma membrane domain was detected (see Figure 8 and Supplementary Figure S9). In fact, we observed punctate structures resembling tight junctions between neighboring cells in all three experimental groups, namely, euthyroid, hypothyroid, and hyperthyroid mice. These findings indicate that lateral cell–cell junctions and the polarized organization of thyrocytes remained largely preserved in the experimental mouse models used in this study. Taken together, these observations argue against the presence of autophagy or overt cytotoxicity in the models investigated.

Ideally, TH supplementation to restore thyroid function in hypothyroid models would provide valuable additional insights. Such approaches have been pursued by others [48,49,50]. However, a meaningful substitution experiment should include T4 alone, T3 alone, and a combination of both hormones to distinguish the causes of the structural and functional thyroid phenotypes associated with hypothyroidism. Such supplementation studies are challenging to design because, in addition to achieving physiologically appropriate circulating T4/T3 ratios, one must also consider the contribution of TH derivatives, such as 3-T1AM, which may be required for the complete restoration of euthyroid conditions. These factors are rarely assessed and often remain inadequately controlled [51]. Because deficiency in the 3-T1AM receptor Taar1 alters HPT axis regulation and induces structural and functional changes in the mouse thyroid gland [52], it can be inferred that dietary supplementation or hormone replacement strategies are far from straightforward.

For the present study, we conclude that downregulation of the TH transporter Mct8 contributes substantially to the phenotypic alterations observed in thyroid gland tissue from mice with experimentally induced hypothyroidism. Thus, little, if any, TH supply from the circulation likely reached the thyroid tissue in these models. Under such conditions, the thyroid gland becomes hypothyroid both intrinsically and extrinsically, while no overt signs of thyrocyte stress were detected (see above). This may explain why other models of hypothyroidism, including hyt/*hyt* mice [53], Dicer knockout mice [45], NIS knockout mice [46], and Tg-mutant mice [47,49,50], display thyroid tissue phenotypes that differ from those observed in the present study.

## 4. Materials and Methods

### 4.1. Animals with Thyroxine-Induced Hyperthyroidism or Hypothyroidism and Tissue Sampling

Two-month-old male wild-type C57BL/6JRj mice (Janvier Labs, Le Genest Saint Isle, France) were housed under standard conditions in the animal facility of the University Hospital Essen, University of Duisburg-Essen, Germany, as described previously [8,9]. In brief, mice were kept in groups of three to five animals at room temperature (23 ± 1 °C) under a 12:12 h light–dark cycle. Water and standard diet were provided ad libitum.

To induce hyperthyroidism (high TH), six mice received T4 in drinking water [1 mg/mL, Sigma-Aldrich (T2376), USA; stock solution: 100 μg/mL of T4 dissolved in 40 mM of NaOH and 2 g/L of bovine serum albumin] over a period of 4 weeks. To induce hypothyroidism (low TH), eight mice received a low-iodine diet (LoI; MD.1571, Envigo, Indianapolis, IN, USA) and drinking water supplemented with 0.04% methimazole [MMI, Sigma-Aldrich (301507), St. Louis, MO, USA], 0.5% sodium perchlorate [ClO_4_^−^, Sigma-Aldrich (310514), St. Louis, MO, USA], and 0.3% saccharine as sweetener [Sigma-Aldrich (240931), St. Louis, MO, USA] (LoI/MMI/ClO_4_^−^) over a period of 4 weeks. The eight euthyroid control animals received a normal diet (MD.1572, Envigo, Indianapolis, IN, USA) and drinking water without supplements. Thyroid states were determined accordingly and as previously reported [8,9].

Thyroid tissue sampling was performed as previously described [10,11]. Thyroid glands for biochemical analysis were detached from the trachea and shock-frozen in liquid nitrogen and stored at −80 °C; numbers of animals were: *n* = 5 (eu), 3 (hyper), and 5 (hypo). For morphological analysis, thyroid glands from three animals per thyroid condition attached to the trachea were fixed in 4% paraformaldehyde in 200 mM Hepes, pH 7.4. All thyroid tissue samples were transported to Constructor University Bremen for further analysis.

### 4.2. Cryo-Sectioning

Thyroid glands remaining attached to the trachea were cryo-sectioned as previously described [10,11]; numbers of animals were: *n* = 3, 3, and 3 per thyroid condition. Preparation of thyroid lobes for biochemical analyses was performed as described in the same studies [10,11]; numbers of animals were: *n* = 5, 3, and 5 per thyroid condition.

### 4.3. Indirect Immunofluorescence and Phalloidin Staining

Thyroid tissue sections mounted on microscopic slides were processed as described previously [10,11]. The F-actin cytoskeleton was visualized by phalloidin staining, whereas indirect immunofluorescence staining was performed using the primary antibodies listed in Table 2. Negative controls incubated with secondary antibodies only showed no detectable staining (Supplementary Figure S11). Numbers of animals were: *n* = 3, 3, and 3 per thyroid condition, except *n* = 3, 2, and 3 per condition for cathepsin B and L staining.

### 4.4. Image Acquisition

Collagen IV-, CMO-, and Mct8-stained thyroid tissue sections were imaged using a Zeiss LSM 510 META laser scanning microscope (Carl Zeiss Microscopy GmbH, Oberkochen, Germany), and images were taken with a pinhole opening of 1 Airy unit and at a resolution of 1024 × 1024 pixels and exported in TIFF format using LSM 5 software (version 3.2). For standardized comparisons, special care was taken to use identical laser and histogram settings for images from different thyroid states. In addition, the tissue image cytometry setup using a TissueFAXS SL+ (TissueFAXS 6.0.6245.136 version, TissueGnostics GmbH, Vienna, Austria) whole-slide imager was used, and images were acquired at a resolution of 2048 × 2048 pixels prior to image stitching, tissue segmentation, and image analysis with the StrataQuest 8.0.85 software (TissueGnostics GmbH, Vienna, Austria). Laminin- and Ki67-stained thyroid tissue sections were imaged using a Zeiss LSM980 Airyscan 2 laser scanning microscope (Carl Zeiss Microscopy GmbH, Oberkochen, Germany) at a resolution of 2492 × 2492 pixels. F-actin and laminin microscopy images were acquired at 5 positions in z, with 1 µm spacing, displayed as extended-focus images and exported in TIFF format using LSM Zen lite (version 3.9). For standardized comparisons, special care was taken to use identical laser power and detector settings for samples from different thyroid conditions. The number of biological replicates per experimental condition used in this study and the number of thyroid follicles or micrographs analyzed are mentioned in the respective figure legends.

### 4.5. Morphological Image Analysis of Thyroid Glands by CellProfiler™ and by StrataQuest

The EExt, thyroid follicle and luminal areas from *n* = 2, 2, 3 per thyroid condition were analyzed with the open-source software CellProfiler™ 3.1.9 [12] following our established pipeline described before [10,11] and as detailed in Supplementary Figure S1. In brief, PFA-fixed thyroid tissue was cryo-sectioned and stained with Draq5™, CellMaskOrange™, and antibodies against collagen IV to visualize the nuclei, cytoplasm, and the ECM, respectively. All channels of microscopy images were merged and converted into grayscale images to identify the follicle lumen. The identified lumen areas served as seeds that were propagated toward the collagen IV-positive basal lamina surrounding individual follicles. Subtracting the lumen area from the respective follicle area yielded the epithelial extension of the monolayers. The resulting spreadsheet file, listing the lumen areas, follicle areas and EExt per image, was subsequently imported into GraphPad Prism (version 8.0.2 for Windows, GraphPad Software, Boston, MA, USA) for statistical analysis and data visualization. The StrataQuest 8.0.85 software was used to generate an analysis pipeline for the samples acquired by TissueFAXS SL+ platform for detailed analysis of tissue characteristics at the cellular level. The lumen seed was identified based on the microscopy image channels (green—FITC—ECM; red—A546—CMO; and blue—Draq5—nuclei). Luminal areas surrounded by red-stained areas were added to isolated red areas with high compactness. These were separated based on the presence of green—FITC—ECM to generate the individual seeds. These were propagated outward for automatic follicle detection. Finally, manual correction of follicles was performed where necessary. For each follicle, the following were calculated: area (μm^2^) of lumen, area (μm^2^) and intensity of red—A546—CMO, area (μm^2^) and intensity of green—FITC—ECM inside and around the detected follicle area, and distance of follicle from the thyroid lobe edge. Finally, raw data for each follicle was exported.

### 4.6. Tissue Lysate Preparation

Snap-frozen lobes of thyroid glands were lysed and lysates were cleared [10] before protein concentrations were determined [54]. Protein lysates were denatured in sample buffer containing 50 mM Tris-HCl (pH 7.6), 2.5% sodium dodecyl sulfate (SDS), 125 mM dithiothreitol, and 0.04% (*w*/*v*) bromophenol blue for 5 min at 95 °C and stored at −20 °C until SDS-PAGE. Numbers of animals analyzed were: *n* = 5, 3, and 5 per thyroid condition.

### 4.7. SDS-PAGE, Immunoblotting, and Silver Staining

Protein lysates were separated by SDS-PAGE on horizontal SDS Gradient 8–18 ExcelGel™ gels (GE Healthcare, via VWR International GmbH, Darmstadt, Germany) along with a Page Ruler Prestained Protein ladder (Thermo Fisher Scientific, Bremen, Germany, #26616), transferred onto nitrocellulose membranes (#HP40.1, Carl Roth GmbH, Karlsruhe, Germany) by semi-dry blotting, and immunostained with antibodies specific for cathepsins B, D, L, and Mct8 diluted in PBS-T (see Table 2) [10]. Proteins were visualized by chemiluminescence ECL Western Blotting substrate on CL-XPosure™ films (Pierce via Thermo Scientific, Schwerte, Germany). Band densitometry analysis of anti-cathepsin B-, D-, L-, and Mct8-positive bands was performed using Image Studio Lite version 5.2 (LI-COR Biosciences GmbH, Bad Homburg, Germany).

A total of 0.5 μg of protein from thyroid lysate preparations was separated on horizontal SDS Gradient 8-18 ExcelGel™ gels (GE Healthcare, via VWR International GmbH, Darmstadt, Germany), and gels were silver-stained [55]. Densitometry analysis was conducted as described above.

Full-length blots and gels are presented throughout the manuscript; uncropped blots and gels as well as secondary antibody controls have been deposited in the journal’s repository. Numbers of animals analyzed regarding Tg status were: *n* = 5, 3, and 5 per condition. Numbers of animals regarding cathepsins and Mct8 were: *n* = 4, 3, and 5 per condition.

### 4.8. Measurement of Laminin Signals from Immunofluorescence Images by CellProfiler™

Mean laminin fluorescence intensity signals of microscopic images from euthyroid, hyperthyroid, and hypothyroid tissue of three animals per thyroid condition were measured using CellProfiler™ 4.2.8 [12]. Microscopy images exported as TIFF files of laminin- and Draq5™-stained thyroid tissue sections were imported into CellProfiler™, and a pipeline combining different image analysis modules was established. First, the ‘ColorToGray’ module splits the RGB TIFF file input image into respective channels, i.e., green (laminin) and red (Draq5™). The resulting grayscale images were used to measure the mean image intensity from the green channel, representing laminin, and to count Draq5™-stained cells (used for normalization), based on fluorescence intensities, typical diameter settings for nuclei (70–250 pixels), and intensity thresholds by using the ‘MeasureImageIntensity’ and ‘IdentifyPrimaryObjects’ modules. The resulting spreadsheet file listing the counted Draq5™ numbers and the mean laminin intensities per image was subsequently imported into GraphPad Prism (version 8.0.2) for statistical analysis and data visualization.

### 4.9. Determination of Proliferation Rates by CellProfiler™

Determination of the proliferation rates of thyrocytes in euthyroid, hyperthyroid, and hypothyroid tissue of three animals per condition was performed using the open-source image analysis software CellProfiler™ 4.2.8 [12]. Microscopy images exported as TIFF files of Ki67- and Draq5™-stained thyroid tissue sections were imported into CellProfiler™, and a pipeline combining different image analysis modules was established. First, the ‘ColorToGray’ module splits the RGB TIFF file input image into respective channels, i.e., green (Ki67) and red (Draq5™). The resulting grayscale images were used to identify Ki67-positive and Draq5™-positive nuclei using fluorescence intensity thresholds and expected nuclear diameters (70–250 pixels) by using ‘IdentifyPrimaryObjects’ modules, while ‘EditObjectsManually’ was used to eliminate false positive objects. The resulting spreadsheet file listing the counted Draq5™- and Ki67-positive nuclei per image was subsequently imported into GraphPad Prism (version 8.0.2) for statistical analysis and data visualization.

### 4.10. Statistical Analysis

Data are presented as means ± or + standard deviation (SD). Densitometric measurements from immunopositive bands were normalized to the total protein loading per lane. For all quantitative analyses, changes relative to euthyroid controls were calculated.

For morphometry data, normality was assessed using the Shapiro–Wilk test. When groups violated the assumption of normality, the primary analysis was conducted using a Kruskal–Wallis test followed by Dunn’s post hoc comparisons with Holm correction for multiple testing. To evaluate the robustness of the findings to the choice of statistical method, a one-way analysis of variance (ANOVA) followed by Dunnett’s multiple-comparison post hoc test was additionally performed as a sensitivity analysis using GraphPad Prism™ (version 8.0.2; GraphPad Software, Boston, MA, USA). Concordance between the non-parametric and parametric approaches was used to assess the stability of the observed group differences. Adjusted *p*-values are reported for comparisons of hyperthyroid versus euthyroid and hypothyroid versus euthyroid groups. Differences were considered statistically significant at *p* < 0.05.

## 5. Conclusions

In conclusion, the findings of this study (see Graphical Abstract and Table 1) suggest that cathepsin L expression is upregulated in both T4-induced exogenous hyperthyroidism and hypothyroidism. Moreover, cathepsin L appears to be regulated at the (post)translational level in hypothyroidism, as evidenced by the increased abundance of its mature single-chain and heavy-chain forms. The elevated cathepsin L levels may explain its localization within the follicular lumen and its re-association with the apical plasma membrane of thyrocytes in hypothyroid tissue, whereas cathepsin B was downregulated. By contrast, cathepsin D remained unaffected under conditions of elevated circulating TSH concentrations (hypothyroidism) but was downregulated under low-TSH conditions (hyperthyroidism). Together, these observations point to a specific role for cathepsin L in thyroid dysfunction and may be linked to its established functions in the immune system, particularly its involvement in endosomal antigen processing [56].

Interestingly, Tg itself may contribute to the self-regulation of thyroid function and Tg processing, as its structure contains thyropin domains that can inhibit the proteases responsible for degrading their natural substrate, Tg [7,23,32,57]. Furthermore, Tg processing and degradation for TH liberation are initiated within the follicular lumen and continue following Tg internalization along the endocytic pathway [7,18,25]. Thus, thyroid self-regulation must be viewed as a highly integrated process involving follicular, cellular, and sub-cellular mechanisms that operate alongside classical TSH-mediated control.

Overall, our findings suggest that chronic thyroid dysfunction, particularly hypothyroidism, promotes alterations in thyrocytes. Future studies are needed to clarify the significance of specific proteases, such as cathepsin L, in self-thyrotoxic processes associated with markedly reduced Mct8 protein levels. Mechanistic investigations would benefit from advanced experimental systems, including thyroid organoids, thyroid organ-on-a-chip models, and/or mouse models of thyroid cancer [58,59,60].

## Figures and Tables

**Table 1:** Summary of the main findings in this study.

	Hyperthyroid	Hypothyroid
Tg-dimeric	−65%	−92%
Tg-monomeric	n.s.	−86%
Tg-fragments	n.s.	+77%
Cath B—pro	−62%	−41%
Cath B—SC	−84%	−53%
Cath B—HC	−70%	−63%
Cath B localization	endo-lysosomes (=)	endo-lysosomes (=)
Cath D—pro	−79%	n.s.
Cath D—SC	n.s.	n.s.
Cath D localization	endo-lysosomes (=)	endo-lysosomes (=)
Cath L—pro	+ 261%	+ 1084%
Cath L—SC	n.s.	+254%
Cath L—HC	n.s.	+152%
Cath L localization	endo-lysosomes (=)	intracellular (↑), apical PM (↑)
Mct8—dimeric	n.s.	−85%
Mct8—monomeric	n.s.	n.s.
Mct8 localization	basolateral (=)	basolateral (=)
TC death (luminal remnants)	n.s.	n.s.
TC proliferation (Ki67)	n.s.	+1614%
Laminin localization	BL/ECM (=)	intracellular (↑), distorted follicle shapes
Laminin IF signal intensity	n.s.	+272%

**Table 2:** List of primary antibodies used in this study.

Antigen	Biological Source	Species Reactivity	Company/ Provider	Catalog No.	Dilution in IIF ^1^	Dilution in IB ^1^
Cathepsin B	goat	mouse	Neuromics, through Acris, Herford, Germany	GT15057	1:100	1:1000
Cathepsin D	rabbit	human	Calbiochem, through Merck-Millipore, Darmstadt, Germany	IM-16	1:10	1:500
Cathepsin L	goat	mouse	Neuromics, through Acris, Herford, Germany	GT15049	1:100	1:1000
Mct8	rabbit	human	Atlas Antibodies, Stockholm, Sweden	HPA072719	1:80	1:500
Ki67 [SP6]	rabbit	human	Abcam, Cambridge, UK	ab16667	1:50	n.a.
Laminin	rabbit	human	Abcam, Cambridge, UK	ab11575	1:50	n.a.
Collagen IV	rabbit	mouse	Novotech, through BioLogo, Kronshagen, Germany	CO20451	1:40	n.a.

^1^ IIF, indirect immunofluorescence; IB, immunoblotting.

## Data Availability

The original contributions presented in this study are included in the article/Supplementary Material. Further inquiries can be directed to the corresponding author.

## Supplementary Materials

The following supporting information can be downloaded at: https://www.mdpi.com/article/10.3390/ijms27209345/s1.

## Abbreviations

The following abbreviations are used in this manuscript:

CMO	CellMaskOrange™
ECM	extracellular matrix
EExt	epithelial extension
EMT	epithelial-to-mesenchymal transition
ER	endoplasmic reticulum
GD	Graves’ Disease
HC	heavy chain
HPT	hypothalamic–pituitary–thyroid
HRT	hormone replacement therapy
Lat2	L-type amino acid transporter 2
Mct8	monocarboxylate transporter 8
MMI	methimazole
T4	thyroxine
Taar1	trace amine-associated receptor 1
Tg	thyroglobulin
TH	thyroid hormones
TPO	thyroid peroxidase
TSH	thyroid-stimulating hormone
SC	single chain
