# Supplementary material for: Hypothyroidism and Thyroxine-Induced Hyperthyroidism Differentially Affect Cathepsins, Mct8, and Thyroid Tissue Architecture in Male Mice
Source: Int J Mol Sci. 2026 Oct 21;27(20):9345. doi: 10.3390/ijms27209345 (PMC13705438; doi:10.3390/ijms27209345)
Supplement: Supplementary file 1 [file ijms-27-09345-s001.zip › ijms-4414128-supplementary.pdf]

Hypothyroidism and Thyroxine-Induced Hyperthyroidism Differentially Affect Cathepsins, Mct8, and Thyroid Tissue Architecture in Male Mice

Maren Rehders, Helena Kerp, Robert Nica, Rupert Ecker, Dagmar Führer and Klaudia Brix

Supplementary Figure S1: Cell Profiler™ pipeline settings for morphological analysis of thyroid tissue.

CellProfiler Pipeline (http://www.cellprofiler.org) Eu_Hyper_Hypo_Morphology
Version: 5, DateRevision:428
GitHash: ModuleCount:41
Images:[module_num:1|svn_version:'Unknown'|variable_revision_number:2|show_window:True|notes:['To begin creating your project, use the Images module to compile a list of files and/or folders that you want to analyze. You can also specify a set of rules to include only the desired files in your selected folders.', '---', 'Settings converted from legacy pipeline.']|batch_state:array([], dtype=uint8)|enabled:True|wants_pause:False]
:
Filter images?:Custom
Select the rule criteria:or (or (file does contain "_a"))
Metadata:[module_num:2|svn_version:'Unknown'|variable_revision_number:6|show_window:True|notes:['The Metadata module optionally allows you to extract information describing your images (i.e, metadata) which will be stored along with your measurements. This information can be contained in the file name and/or location, or in an external file.', '---', 'Settings converted from legacy pipeline.']|batch_state:array([], dtype=uint8)|enabled:True|wants_pause:False]
Extract metadata?:Yes
Metadata data type:Text
Metadata types:{}
Extraction method count:1
Metadata extraction method:Extract from file/folder names
Metadata source:File name
Regular expression to extract from file name:^(?P<Plate>.*)_(?P<Well>[A-P][0-9]{2})_s(?P<Site>[0-9])
Regular expression to extract from folder name:(?P<Date>[0-9]{4}_[0-9]{2}_[0-9]{2})$
Extract metadata from:All images
Select the filtering criteria:and (file does contain "")
Metadata file location:Elsewhere...|C:\\Users\\mrehders\\Desktop\\Output
Match file and image metadata:[]
Use case insensitive matching?:No
Metadata file name:Nuclei.csv
Does cached metadata exist?:No

NamesAndTypes:[module_num:3|svn_version:'Unknown'|variable_revision_number:8|show_window:True|notes:[' The NamesAndTypes module allows you to assign a meaningful name to each image by which other modules will refer to it.', '---', 'Settings converted from legacy pipeline.', 'Please change any color images from "Load as Grayscale image" to "Load as Color image"']|batch_state:array([], dtype=uint8)|enabled:True|wants_pause:False]
Assign a name to:All images
Select the image type:Color image
Name to assign these images:OrigColor
Match metadata:[]
Image set matching method:Order
Set intensity range from:Image metadata
Assignments count:1
Single images count:0
Maximum intensity:255.0
Process as 3D?:No
Relative pixel spacing in X:1.0
Relative pixel spacing in Y:1.0
Relative pixel spacing in Z:1.0
Select the rule criteria:and (image does iscolor)
Name to assign these images:OrigColor
Name to assign these objects:Cell
Select the image type:Color image
Set intensity range from:Image metadata
Maximum intensity:255.0

Groups:[module_num:4|svn_version:'Unknown'|variable_revision_number:2|show_window:True|notes:['The Groups module optionally allows you to split your list of images into image subsets (groups) which will be processed independently of each other. Examples of groupings include screening batches, microtiter plates, time-lapse movies, etc.', '---', 'Settings converted from legacy pipeline.']|batch_state:array([], dtype=uint8)|enabled:True|wants_pause:False]
Do you want to group your images?:No

```
    grouping metadata count:1
    Metadata category:None

ColorToGray:[module_num:5|svn_version:'Unknown'|variable_revision_number:4|show_window:True|notes:[]|batc
h_state:array([], dtype=uint8)|enabled:True|wants_pause:False]
    Select the input image:OrigColor
    Conversion method:Split
    Image type:RGB
    Name the output image:OrigGray
    Relative weight of the red channel:1
    Relative weight of the green channel:1
    Relative weight of the blue channel:1
    Convert red to gray?:Yes
    Name the output image:OrigRed
    Convert green to gray?:Yes
    Name the output image:OrigGreen
    Convert blue to gray?:Yes
    Name the output image:OrigBlue
    Convert hue to gray?:Yes
    Name the output image:OrigHue
    Convert saturation to gray?:Yes
    Name the output image:OrigSaturation
    Convert value to gray?:Yes
    Name the output image:OrigValue
    Channel count:1
    Channel number:1
    Relative weight of the channel:1
    Image name:Channel1

ColorToGray:[module_num:6|svn_version:'Unknown'|variable_revision_number:4|show_window:True|notes:['Use
the grayscale version of the image to find the space within the follicle.']|batch_state:array([],
dtype=uint8)|enabled:True|wants_pause:False]
    Select the input image:OrigColor
    Conversion method:Combine
    Image type:RGB
    Name the output image:OrigGray
    Relative weight of the red channel:1
    Relative weight of the green channel:1
    Relative weight of the blue channel:1
    Convert red to gray?:No
    Name the output image:OrigRed
    Convert green to gray?:Yes
    Name the output image:OrigGreen
    Convert blue to gray?:Yes
    Name the output image:OrigBlue
    Convert hue to gray?:Yes
    Name the output image:OrigHue
    Convert saturation to gray?:Yes
    Name the output image:OrigSaturation
    Convert value to gray?:Yes
    Name the output image:OrigValue
    Channel count:1
    Channel number:1
    Relative weight of the channel:1
    Image name:Channel1

Threshold:[module_num:7|svn_version:'Unknown'|variable_revision_number:12|show_window:True|notes:[]|batch
_state:array([], dtype=uint8)|enabled:True|wants_pause:False]
    Select the input image:OrigGreen
    Name the output image:ThreshOrigGreen_bin1
    Threshold strategy:Global
    Thresholding method:Robust Background
    Threshold smoothing scale:1.3488
    Threshold correction factor:0.5
    Lower and upper bounds on threshold:0.05,1.0
    Manual threshold:0.5
```

Select the measurement to threshold with:None
Two-class or three-class thresholding?:Two classes
Log transform before thresholding?:No
Assign pixels in the middle intensity class to the foreground or the background?:Background
Size of adaptive window:10
Lower outlier fraction:0.05
Upper outlier fraction:0.05
Averaging method:Mean
Variance method:Standard deviation
# of deviations:2
Thresholding method:Default

Threshold:[module_num:8|svn_version:'Unknown'|variable_revision_number:12|show_window:True|notes:['IMPORTANT: Depending on thet background of your CMO staining you have to adjust the lower threshold (in the case of perfect CMO staining use 0.0001, if the lumen/Tg is stained then go up to 0.01 or even 0.1). Ideally you will not have signal in the lumen (!) but clear edges at the apical plasma membrane domain of the epithelial cells. Rather use overexposed CMO and a amplifier offset during imaging to find the balance between blank lumen and nicley stained epithelium.']|batch_state:array([], dtype=uint8)|enabled:True|wants_pause:False]
Select the input image:OrigGray
Name the output image:AllTissue
Threshold strategy:Global
Thresholding method:Manual
Threshold smoothing scale:0
Threshold correction factor:2.0
Lower and upper bounds on threshold:0.1,1.0
Manual threshold:0.1
Select the measurement to threshold with:None
Two-class or three-class thresholding?:Three classes
Log transform before thresholding?:No
Assign pixels in the middle intensity class to the foreground or the background?:Background
Size of adaptive window:10
Lower outlier fraction:0.02
Upper outlier fraction:0.02
Averaging method:Mode
Variance method:Standard deviation
# of deviations:0
Thresholding method:Default

Morph:[module_num:9|svn_version:'Unknown'|variable_revision_number:5|show_window:True|notes:[]|batch_state:array([], dtype=uint8)|enabled:True|wants_pause:False]
Select the input image:ThreshOrigGreen_bin1
Name the output image:ThreshOrigGreen_bin2
Select the operation to perform:bridge
Number of times to repeat operation:Custom
Repetition number:2
Rescale values from 0 to 1?:Yes
Select the operation to perform:clean
Number of times to repeat operation:Forever
Repetition number:2
Rescale values from 0 to 1?:Yes
Select the operation to perform:fill
Number of times to repeat operation:Forever
Repetition number:2
Rescale values from 0 to 1?:Yes

Morph:[module_num:10|svn_version:'Unknown'|variable_revision_number:5|show_window:True|notes:[]|batch_state:array([], dtype=uint8)|enabled:True|wants_pause:False]
Select the input image:ThreshOrigGreen_bin2
Name the output image:ThreshOrigGreen_bin
Select the operation to perform:skelpe
Number of times to repeat operation:Once
Repetition number:3
Rescale values from 0 to 1?:Yes
Select the operation to perform:bridge
Number of times to repeat operation:Forever
Repetition number:4

Rescale values from 0 to 1?:Yes
Select the operation to perform:clean
Number of times to repeat operation:Once
Repetition number:2
Rescale values from 0 to 1?:Yes
Select the operation to perform:thicken
Number of times to repeat operation:Custom
Repetition number:2
Rescale values from 0 to 1?:Yes

ImageMath:[module_num:11|svn_version:'Unknown'|variable_revision_number:5|show_window:True|notes:['Since foreground/background detection depends on the foreground being lighter than the background, invert the image intensities. ']|batch_state:array([], dtype=uint8)|enabled:True|wants_pause:False]
Operation:Invert
Raise the power of the result by:1
Multiply the result by:1
Add to result:0
Set values less than 0 equal to 0?:Yes
Set values greater than 1 equal to 1?:Yes
Replace invalid values with 0?:Yes
Ignore the image masks?:No
Name the output image:InvertedGray
Image or measurement?:Image
Select the first image:AllTissue
Multiply the first image by:1
Measurement:
Image or measurement?:Image
Select the second image:
Multiply the second image by:1
Measurement:

IdentifyPrimaryObjects:[module_num:12|svn_version:'Unknown'|variable_revision_number:15|show_window:True|notes:["Identify the dark spaces. Some adjustment of the smoothing/maxima separation paremeters was needed to identify the isolated spaces as a single unit. However, this identifies the follcile spaces as well as low-contrast regions which are not part of the follicle. Alos, I'm keeping only the objects not touching the image border, since per-object morphological measurements would be inaccurate otherwise."]|batch_state:array([], dtype=uint8)|enabled:True|wants_pause:False]
Select the input image:InvertedGray
Name the primary objects to be identified:AllDarkSpaces
Typical diameter of objects, in pixel units (Min,Max):20,600
Discard objects outside the diameter range?:Yes
Discard objects touching the border of the image?:Yes
Method to distinguish clumped objects:Shape
Method to draw dividing lines between clumped objects:Shape
Size of smoothing filter:35
Suppress local maxima that are closer than this minimum allowed distance:50
Speed up by using lower-resolution image to find local maxima?:Yes
Fill holes in identified objects?:After both thresholding and declumping
Automatically calculate size of smoothing filter for declumping?:No
Automatically calculate minimum allowed distance between local maxima?:No
Handling of objects if excessive number of objects identified:Continue
Maximum number of objects:500
Use advanced settings?:Yes
Threshold setting version:12
Threshold strategy:Global
Thresholding method:Manual
Threshold smoothing scale:1.3488
Threshold correction factor:1
Lower and upper bounds on threshold:0.30000,1.000000
Manual threshold:0.5
Select the measurement to threshold with:None
Two-class or three-class thresholding?:Two classes
Log transform before thresholding?:No
Assign pixels in the middle intensity class to the foreground or the background?:Background
Size of adaptive window:10
Lower outlier fraction:0.05

```
    Upper outlier fraction:0.05
    Averaging method:Mean
    Variance method:Standard deviation
    # of deviations:2
    Thresholding method:Default

MeasureObjectSizeShape:[module_num:13|svn_version:'Unknown'|variable_revision_number:3|show_window:True|notes:['Measure the morphology of these spaces in order to identify a morphology-based cutoff.']|batch_state:array([], dtype=uint8)|enabled:True|wants_pause:False]
    Select object sets to measure:AllDarkSpaces
    Calculate the Zernike features?:No
    Calculate the advanced features?:No

DisplayDataOnImage:[module_num:14|svn_version:'Unknown'|variable_revision_number:6|show_window:True|notes:['Form factor is a measure of cirularity; the closer to 1, the more circular the object is.']|batch_state:array([], dtype=uint8)|enabled:True|wants_pause:False]
    Display object or image measurements?:Object
    Select the input objects:AllDarkSpaces
    Measurement to display:AreaShape_FormFactor
    Select the image on which to display the measurements:InvertedGray
    Text color:#24DB2E
    Name the output image that has the measurements displayed:DisplayLumenFilter
    Font size (points):10
    Number of decimals:2
    Image elements to save:Image
    Annotation offset (in pixels):0
    Display mode:Text
    Color map:jet
    Display background image?:Yes
    Color map scale:Use this image\'s measurement range
    Color map range:0.0,1.0
    Font:Agency FB
    Use scientific notation?:No
    Font weight:normal

FilterObjects:[module_num:15|svn_version:'Unknown'|variable_revision_number:10|show_window:True|notes:['Filter the objects on the basis of form factor.']|batch_state:array([], dtype=uint8)|enabled:True|wants_pause:False]
    Select the objects to filter:AllDarkSpaces
    Name the output objects:Lumen1
    Select the filtering mode:Measurements
    Select the filtering method:Limits
    Select the objects that contain the filtered objects:None
    Select the location of the rules or classifier file:Elsewhere...|C:\\Users\\jweber\\Desktop\\Desktop Jonas\\CellProfiler Test\\cp test
    Rules or classifier file name:rules.txt
    Class number:1
    Measurement count:1
    Additional object count:0
    Assign overlapping child to:Both parents
    Keep removed objects as a separate set?:No
    Name the objects removed by the filter:RemovedObjects
    Select the measurement to filter by:AreaShape_FormFactor
    Filter using a minimum measurement value?:Yes
    Minimum value:0.2
    Filter using a maximum measurement value?:No
    Maximum value:1
    Allow fuzzy feature matching?:No

EditObjectsManually:[module_num:16|svn_version:'Unknown'|variable_revision_number:4|show_window:True|notes:['Quality check point!']|batch_state:array([], dtype=uint8)|enabled:True|wants_pause:False]
    Select the objects to be edited:Lumen1
    Name the edited objects:Lumen
    Numbering of the edited objects:Renumber
    Display a guiding image?:Yes
    Select the guiding image:OrigGray
    Allow overlapping objects?:No
```

OverlayOutlines:[module_num:17|svn_version:'Unknown'|variable_revision_number:4|show_window:True|notes:[]|batch_state:array([], dtype=uint8)|enabled:True|wants_pause:False]
Display outlines on a blank image?:No
Select image on which to display outlines:OrigColor
Name the output image:OrigOverlayLumen
Outline display mode:Color
Select method to determine brightness of outlines:Max of image
How to outline:Inner
Select outline color:white
Select objects to display:AllDarkSpaces

ImageMath:[module_num:18|svn_version:'Unknown'|variable_revision_number:5|show_window:True|notes:['Use the CMO-staining and substract the binary threshold from step6 (Green) to outline borders of follicles. Use to identify secondary objects.']|batch_state:array([], dtype=uint8)|enabled:True|wants_pause:False]
Operation:Subtract
Raise the power of the result by:1.0
Multiply the result by:1.0
Add to result:0.0
Set values less than 0 equal to 0?:Yes
Set values greater than 1 equal to 1?:Yes
Replace invalid values with 0?:Yes
Ignore the image masks?:No
Name the output image:SubtractGreen
Image or measurement?:Image
Select the first image:AllTissue
Multiply the first image by:1.0
Measurement:
Image or measurement?:Image
Select the second image:ThreshOrigGreen_bin
Multiply the second image by:1.0
Measurement:

IdentifySecondaryObjects:[module_num:19|svn_version:'Unknown'|variable_revision_number:10|show_window:True|notes:['Grow the surviving colloid objects to get the ful follicle.']|batch_state:array([], dtype=uint8)|enabled:True|wants_pause:False]
Select the input objects:Lumen
Name the objects to be identified:Follicle1
Select the method to identify the secondary objects:Distance - B
Select the input image:SubtractGreen
Number of pixels by which to expand the primary objects:38
Regularization factor:0.001
Discard secondary objects touching the border of the image?:No
Discard the associated primary objects?:Yes
Name the new primary objects:FilteredNuclei
Fill holes in identified objects?:No
Threshold setting version:12
Threshold strategy:Global
Thresholding method:Manual
Threshold smoothing scale:1.3488
Threshold correction factor:0.8
Lower and upper bounds on threshold:0.000000,1.000000
Manual threshold:0
Select the measurement to threshold with:None
Two-class or three-class thresholding?:Two classes
Log transform before thresholding?:No
Assign pixels in the middle intensity class to the foreground or the background?:Background
Size of adaptive window:10
Lower outlier fraction:0.05
Upper outlier fraction:0.05
Averaging method:Mean
Variance method:Standard deviation
# of deviations:2
Thresholding method:Default

EditObjectsManually:[module_num:20|svn_version:'Unknown'|variable_revision_number:4|show_window:True|notes:['Quality check point!']|batch_state:array([], dtype=uint8)|enabled:True|wants_pause:False]
Select the objects to be edited:Follicle1
Name the edited objects:Follicle
Numbering of the edited objects:Renumber
Display a guiding image?:Yes
Select the guiding image:OrigGray
Allow overlapping objects?:No

IdentifyTertiaryObjects:[module_num:21|svn_version:'Unknown'|variable_revision_number:3|show_window:True|notes:['Identify the per-follcile epithelium, as compared to before, where the epithelial boudaries were not considered.']|batch_state:array([], dtype=uint8)|enabled:True|wants_pause:False]
Select the larger identified objects:Follicle
Select the smaller identified objects:Lumen
Name the tertiary objects to be identified:Epithelium
Shrink smaller object prior to subtraction?:No

OverlayOutlines:[module_num:22|svn_version:'Unknown'|variable_revision_number:4|show_window:True|notes:[]|batch_state:array([], dtype=uint8)|enabled:True|wants_pause:False]
Display outlines on a blank image?:No
Select image on which to display outlines:OrigGray
Name the output image:EO
Outline display mode:Color
Select method to determine brightness of outlines:Max of image
How to outline:Inner
Select outline color:Green
Select objects to display:Follicle

MeasureObjectSizeShape:[module_num:23|svn_version:'Unknown'|variable_revision_number:3|show_window:True|notes:['Measure the morphology of each follicle.']|batch_state:array([], dtype=uint8)|enabled:True|wants_pause:False]
Select object sets to measure:Lumen, Epithelium, Follicle
Calculate the Zernike features?:No
Calculate the advanced features?:No

RelateObjects:[module_num:24|svn_version:'Unknown'|variable_revision_number:5|show_window:True|notes:['#to relate epithelium as tertiary object to follicles --> important for assigning the correct follicles to the related lumen objects (for #Math steps).']|batch_state:array([], dtype=uint8)|enabled:True|wants_pause:False]
Parent objects:Epithelium
Child objects:Follicle
Calculate child-parent distances?:Minimum
Calculate per-parent means for all child measurements?:No
Calculate distances to other parents?:No
Do you want to save the children with parents as a new object set?:No
Name the output object:None
Parent name:None

CalculateMath:[module_num:25|svn_version:'Unknown'|variable_revision_number:3|show_window:True|notes:['Area calculation: Friedrichs et al 2003 ', 'For calculating the epithelial extension']|batch_state:array([], dtype=uint8)|enabled:True|wants_pause:False]
Name the output measurement:AreaCalc
Operation:Subtract
Select the minuend measurement type:Object
Select the minuend objects:Follicle
Select the minuend measurement:AreaShape_Area
Multiply the above operand by:1.0
Raise the power of above operand by:1.0
Select the subtrahend measurement type:Object
Select the subtrahend objects:Lumen
Select the subtrahend measurement:AreaShape_Area
Multiply the above operand by:1.0
Raise the power of above operand by:1.0
Take log10 of result?:No
Multiply the result by:1
Raise the power of result by:1.0
Add to the result:0.0

How should the output value be rounded?:Not rounded
Enter how many decimal places the value should be rounded to:0
Constrain the result to a lower bound?:No
Enter the lower bound:0.0
Constrain the result to an upper bound?:No
Enter the upper bound:1.0

CalculateMath:[module_num:26|svn_version:'Unknown'|variable_revision_number:3|show_window:True|notes:['Perimter calculation: Friedrichs et al 2003 ', 'For calculating the epithelial extension']|batch_state:array([], dtype=uint8)|enabled:True|wants_pause:False]
Name the output measurement:PerimeterCalc
Operation:Add
Select the first operand measurement type:Object
Select the first operand objects:Follicle
Select the first operand measurement:AreaShape_Perimeter
Multiply the above operand by:1.0
Raise the power of above operand by:1.0
Select the second operand measurement type:Object
Select the second operand objects:Lumen
Select the second operand measurement:AreaShape_Perimeter
Multiply the above operand by:1.0
Raise the power of above operand by:1.0
Take log10 of result?:No
Multiply the result by:1
Raise the power of result by:1.0
Add to the result:0.0
How should the output value be rounded?:Not rounded
Enter how many decimal places the value should be rounded to:0
Constrain the result to a lower bound?:No
Enter the lower bound:0.0
Constrain the result to an upper bound?:No
Enter the upper bound:1.0

CalculateMath:[module_num:27|svn_version:'Unknown'|variable_revision_number:3|show_window:True|notes:['Friedrichs et al 2003 ', 'For calculating the epithelial extension']|batch_state:array([], dtype=uint8)|enabled:True|wants_pause:False]
Name the output measurement:EpithelialExtension
Operation:Divide
Select the numerator measurement type:Object
Select the numerator objects:Follicle
Select the numerator measurement:Math_AreaCalc
Multiply the above operand by:1.0
Raise the power of above operand by:1.0
Select the denominator measurement type:Object
Select the denominator objects:Lumen
Select the denominator measurement:Math_PerimeterCalc
Multiply the above operand by:1.0
Raise the power of above operand by:1.0
Take log10 of result?:No
Multiply the result by:0.5
Raise the power of result by:1.0
Add to the result:0.0
How should the output value be rounded?:Not rounded
Enter how many decimal places the value should be rounded to:0
Constrain the result to a lower bound?:No
Enter the lower bound:0.0
Constrain the result to an upper bound?:No
Enter the upper bound:1.0

CalculateMath:[module_num:28|svn_version:'Unknown'|variable_revision_number:3|show_window:True|notes:[]|batch_state:array([], dtype=uint8)|enabled:True|wants_pause:False]
Name the output measurement:EpithelialExtension_MicroMeter
Operation:None
Select the numerator measurement type:Object
Select the numerator objects:Follicle
Select the numerator measurement:Math_EpithelialExtension

Multiply the above operand by:2.276
Raise the power of above operand by:1.0
Select the second operand measurement type:Image
Select the second operand objects:None
Select the second operand measurement:None
Multiply the above operand by:1.0
Raise the power of above operand by:1.0
Take log10 of result?:No
Multiply the result by:1.0
Raise the power of result by:1.0
Add to the result:0.0
How should the output value be rounded?:Not rounded
Enter how many decimal places the value should be rounded to:0
Constrain the result to a lower bound?:No
Enter the lower bound:0.0
Constrain the result to an upper bound?:No
Enter the upper bound:1.0

CalculateMath:[module_num:29|svn_version:'Unknown'|variable_revision_number:3|show_window:True|notes:[]|batch_state:array([], dtype=uint8)|enabled:True|wants_pause:False]
Name the output measurement:FollicleArea_squareMicroMeter
Operation:None
Select the numerator measurement type:Object
Select the numerator objects:Follicle
Select the numerator measurement:AreaShape_Area
Multiply the above operand by:0.194
Raise the power of above operand by:1.0
Select the second operand measurement type:Image
Select the second operand objects:None
Select the second operand measurement:None
Multiply the above operand by:1.0
Raise the power of above operand by:1.0
Take log10 of result?:No
Multiply the result by:1.0
Raise the power of result by:1.0
Add to the result:0.0
How should the output value be rounded?:Not rounded
Enter how many decimal places the value should be rounded to:0
Constrain the result to a lower bound?:No
Enter the lower bound:0.0
Constrain the result to an upper bound?:No
Enter the upper bound:1.0

CalculateMath:[module_num:30|svn_version:'Unknown'|variable_revision_number:3|show_window:True|notes:[]|batch_state:array([], dtype=uint8)|enabled:True|wants_pause:False]
Name the output measurement:LumenArea_squareMicroMeter
Operation:None
Select the numerator measurement type:Object
Select the numerator objects:Lumen
Select the numerator measurement:AreaShape_Area
Multiply the above operand by:0.194
Raise the power of above operand by:1.0
Select the second operand measurement type:Image
Select the second operand objects:None
Select the second operand measurement:None
Multiply the above operand by:1.0
Raise the power of above operand by:1.0
Take log10 of result?:No
Multiply the result by:1.0
Raise the power of result by:1.0
Add to the result:0.0
How should the output value be rounded?:Not rounded
Enter how many decimal places the value should be rounded to:0
Constrain the result to a lower bound?:No
Enter the lower bound:0.0
Constrain the result to an upper bound?:No

```
    Enter the upper bound:1.0

CalculateMath:[module_num:31|svn_version:'Unknown'|variable_revision_number:3|show_window:True|notes:[]|b
atch_state:array([], dtype=uint8)|enabled:True|wants_pause:False]
    Name the output measurement:EpitheliumArea_squareMicroMeter
    Operation:None
    Select the numerator measurement type:Object
    Select the numerator objects:Epithelium
    Select the numerator measurement:AreaShape_Area
    Multiply the above operand by:0.194
    Raise the power of above operand by:1.0
    Select the second operand measurement type:Image
    Select the second operand objects:None
    Select the second operand measurement:None
    Multiply the above operand by:1.0
    Raise the power of above operand by:1.0
    Take log10 of result?:No
    Multiply the result by:1.0
    Raise the power of result by:1.0
    Add to the result:0.0
    How should the output value be rounded?:Not rounded
    Enter how many decimal places the value should be rounded to:0
    Constrain the result to a lower bound?:No
    Enter the lower bound:0.0
    Constrain the result to an upper bound?:No
    Enter the upper bound:1.0

IdentifyPrimaryObjects:[module_num:32|svn_version:'Unknown'|variable_revision_number:15|show_window:True|
notes:['Identify the nuclei from the blue channel.']|batch_state:array([],
dtype=uint8)|enabled:True|wants_pause:False]
    Select the input image:OrigBlue
    Name the primary objects to be identified:Nuclei
    Typical diameter of objects, in pixel units (Min,Max):12,40
    Discard objects outside the diameter range?:Yes
    Discard objects touching the border of the image?:Yes
    Method to distinguish clumped objects:Intensity
    Method to draw dividing lines between clumped objects:Intensity
    Size of smoothing filter:6
    Suppress local maxima that are closer than this minimum allowed distance:6
    Speed up by using lower-resolution image to find local maxima?:Yes
    Fill holes in identified objects?:After both thresholding and declumping
    Automatically calculate size of smoothing filter for declumping?:No
    Automatically calculate minimum allowed distance between local maxima?:No
    Handling of objects if excessive number of objects identified:Continue
    Maximum number of objects:500
    Use advanced settings?:Yes
    Threshold setting version:12
    Threshold strategy:Global
    Thresholding method:Otsu
    Threshold smoothing scale:1.3488
    Threshold correction factor:0.9
    Lower and upper bounds on threshold:0.20,1.0
    Manual threshold:0.0
    Select the measurement to threshold with:None
    Two-class or three-class thresholding?:Two classes
    Log transform before thresholding?:No
    Assign pixels in the middle intensity class to the foreground or the background?:Background
    Size of adaptive window:10
    Lower outlier fraction:0.05
    Upper outlier fraction:0.05
    Averaging method:Mean
    Variance method:Standard deviation
    # of deviations:2
    Thresholding method:Default
```

```
RelateObjects:[module_num:33|svn_version:'Unknown'|variable_revision_number:5|show_window:True|notes:['Relate the nuclei as children objects to each "parent" follicle; the nuclei whcih overlap a given follicle belong to that follcile.', '', 'Goal #2: One of the readouts of this procedure is the # of nuclei per follicle.']|batch_state:array([], dtype=uint8)|enabled:True|wants_pause:False]
    Parent objects:Epithelium
    Child objects:Nuclei
    Calculate child-parent distances?:Minimum
    Calculate per-parent means for all child measurements?:No
    Calculate distances to other parents?:No
    Do you want to save the children with parents as a new object set?:No
    Name the output object:None
    Parent name:None

MeasureObjectIntensity:[module_num:34|svn_version:'Unknown'|variable_revision_number:4|show_window:True|notes:['Goal #3: Measure the intensity of each follicle from each channel.']|batch_state:array([], dtype=uint8)|enabled:True|wants_pause:False]
    Select images to measure:OrigGreen, OrigBlue, OrigRed
    Select objects to measure:Epithelium

OverlayOutlines:[module_num:35|svn_version:'Unknown'|variable_revision_number:4|show_window:True|notes:['CPF --> Cells per follicle OVERLAY']|batch_state:array([], dtype=uint8)|enabled:True|wants_pause:False]
    Display outlines on a blank image?:No
    Select image on which to display outlines:OrigColor
    Name the output image:CPF
    Outline display mode:Color
    Select method to determine brightness of outlines:Max of image
    How to outline:Inner
    Select outline color:White
    Select objects to display:Follicle
    Select outline color:Blue
    Select objects to display:Follicle

DisplayDataOnImage:[module_num:36|svn_version:'Unknown'|variable_revision_number:6|show_window:True|notes:[]|batch_state:array([], dtype=uint8)|enabled:True|wants_pause:False]
    Display object or image measurements?:Object
    Select the input objects:Lumen
    Measurement to display:Number_Object_Number
    Select the image on which to display the measurements:CPF
    Text color:white
    Name the output image that has the measurements displayed:NR
    Font size (points):12
    Number of decimals:0
    Image elements to save:Image
    Annotation offset (in pixels):0
    Display mode:Text
    Color map:Default
    Display background image?:Yes
    Color map scale:Use this image\'s measurement range
    Color map range:0.0,1.0
    Font:Agency FB
    Use scientific notation?:No
    Font weight:normal

SaveImages:[module_num:37|svn_version:'Unknown'|variable_revision_number:16|show_window:True|notes:[]|batch_state:array([], dtype=uint8)|enabled:True|wants_pause:False]
    Select the type of image to save:Image
    Select the image to save:EO
    Select method for constructing file names:Sequential numbers
    Select image name for file prefix:None
    Enter file prefix:EO
    Number of digits:4
    Append a suffix to the image file name?:No
    Text to append to the image name:
    Saved file format:tiff
    Output file location:Default Input Folder sub-folder|Desktop\\\\Output
    Image bit depth:16-bit integer
```

```
    Overwrite existing files without warning?:No
    When to save:Every cycle
    Record the file and path information to the saved image?:No
    Create subfolders in the output folder?:No
    Base image folder:Elsewhere...|
    How to save the series:T (Time)
    Save with lossless compression?:No

SaveImages:[module_num:38|svn_version:'Unknown'|variable_revision_number:16|show_window:True|notes:[]|batch_state:array([], dtype=uint8)|enabled:True|wants_pause:False]
    Select the type of image to save:Image
    Select the image to save:DisplayLumenFilter
    Select method for constructing file names:Sequential numbers
    Select image name for file prefix:None
    Enter file prefix:DLF
    Number of digits:4
    Append a suffix to the image file name?:No
    Text to append to the image name:
    Saved file format:tiff
    Output file location:Default Input Folder sub-folder|Desktop\\\\Output
    Image bit depth:16-bit integer
    Overwrite existing files without warning?:No
    When to save:Every cycle
    Record the file and path information to the saved image?:No
    Create subfolders in the output folder?:No
    Base image folder:Elsewhere...|
    How to save the series:T (Time)
    Save with lossless compression?:No

SaveImages:[module_num:39|svn_version:'Unknown'|variable_revision_number:16|show_window:True|notes:[]|batch_state:array([], dtype=uint8)|enabled:True|wants_pause:False]
    Select the type of image to save:Image
    Select the image to save:CPF
    Select method for constructing file names:Sequential numbers
    Select image name for file prefix:None
    Enter file prefix:CPF
    Number of digits:4
    Append a suffix to the image file name?:No
    Text to append to the image name:
    Saved file format:tiff
    Output file location:Default Input Folder sub-folder|Desktop\\\\Output
    Image bit depth:16-bit integer
    Overwrite existing files without warning?:No
    When to save:Every cycle
    Record the file and path information to the saved image?:No
    Create subfolders in the output folder?:No
    Base image folder:Elsewhere...|
    How to save the series:T (Time)
    Save with lossless compression?:No

SaveImages:[module_num:40|svn_version:'Unknown'|variable_revision_number:16|show_window:True|notes:[]|batch_state:array([], dtype=uint8)|enabled:True|wants_pause:False]
    Select the type of image to save:Image
    Select the image to save:NR
    Select method for constructing file names:Sequential numbers
    Select image name for file prefix:None
    Enter file prefix:CPF_NR_
    Number of digits:4
    Append a suffix to the image file name?:No
    Text to append to the image name:
    Saved file format:tiff
    Output file location:Default Input Folder sub-folder|Desktop\\\\Output
    Image bit depth:16-bit integer
    Overwrite existing files without warning?:No
    When to save:Every cycle
    Record the file and path information to the saved image?:No
```

```
    Create subfolders in the output folder?:No
    Base image folder:Elsewhere...|
    How to save the series:T (Time)
    Save with lossless compression?:No

ExportToSpreadsheet:[module_num:41|svn_version:'Unknown'|variable_revision_number:13|show_window:True|
notes:[]|batch_state:array([], dtype=uint8)|enabled:True|wants_pause:False]
    Select the column delimiter:Comma (",")
    Add image metadata columns to your object data file?:Yes
    Add image file and folder names to your object data file?:No
    Select the measurements to export:Yes
    Calculate the per-image mean values for object measurements?:Yes
    Calculate the per-image median values for object measurements?:No
    Calculate the per-image standard deviation values for object measurements?:No
    Output file location:Default Input Folder sub-folder|Desktop\\\\Output
    Create a GenePattern GCT file?:No
    Select source of sample row name:Metadata
    Select the image to use as the identifier:None
    Select the metadata to use as the identifier:None
    Export all measurement types?:No
    Press button to select
measurements:Epithelium|AreaShape_Area,Epithelium|Children_Nuclei_Count,Epithelium|Math_EpitheliumArea_
squareMicroMeter,Image|FileName_OrigColor,Lumen|AreaShape_Area,Lumen|Math_LumenArea_squareMicroM
eter,Follicle|AreaShape_FormFactor,Follicle|AreaShape_Area,Follicle|AreaShape_Eccentricity,Follicle|Math_Peri
meterCalc,Follicle|Math_EpithelialExtension,Follicle|Math_EpithelialExtension_MicroMeter,Follicle|Math_FollicleAr
ea_squareMicroMeter
    Representation of Nan/Inf:NaN
    Add a prefix to file names?:No
    Filename prefix:MyExpt_
    Overwrite existing files without warning?:No
    Data to export:Lumen
    Combine these object measurements with those of the previous object?:No
    File name:Lumen_Follicle_Epithelium.csv
    Use the object name for the file name?:No
    Data to export:Follicle
    Combine these object measurements with those of the previous object?:Yes
    File name:DATA.csv
    Use the object name for the file name?:Yes
    Data to export:Epithelium
    Combine these object measurements with those of the previous object?:Yes
    File name:DATA.csv
    Use the object name for the file name?:Yes
```

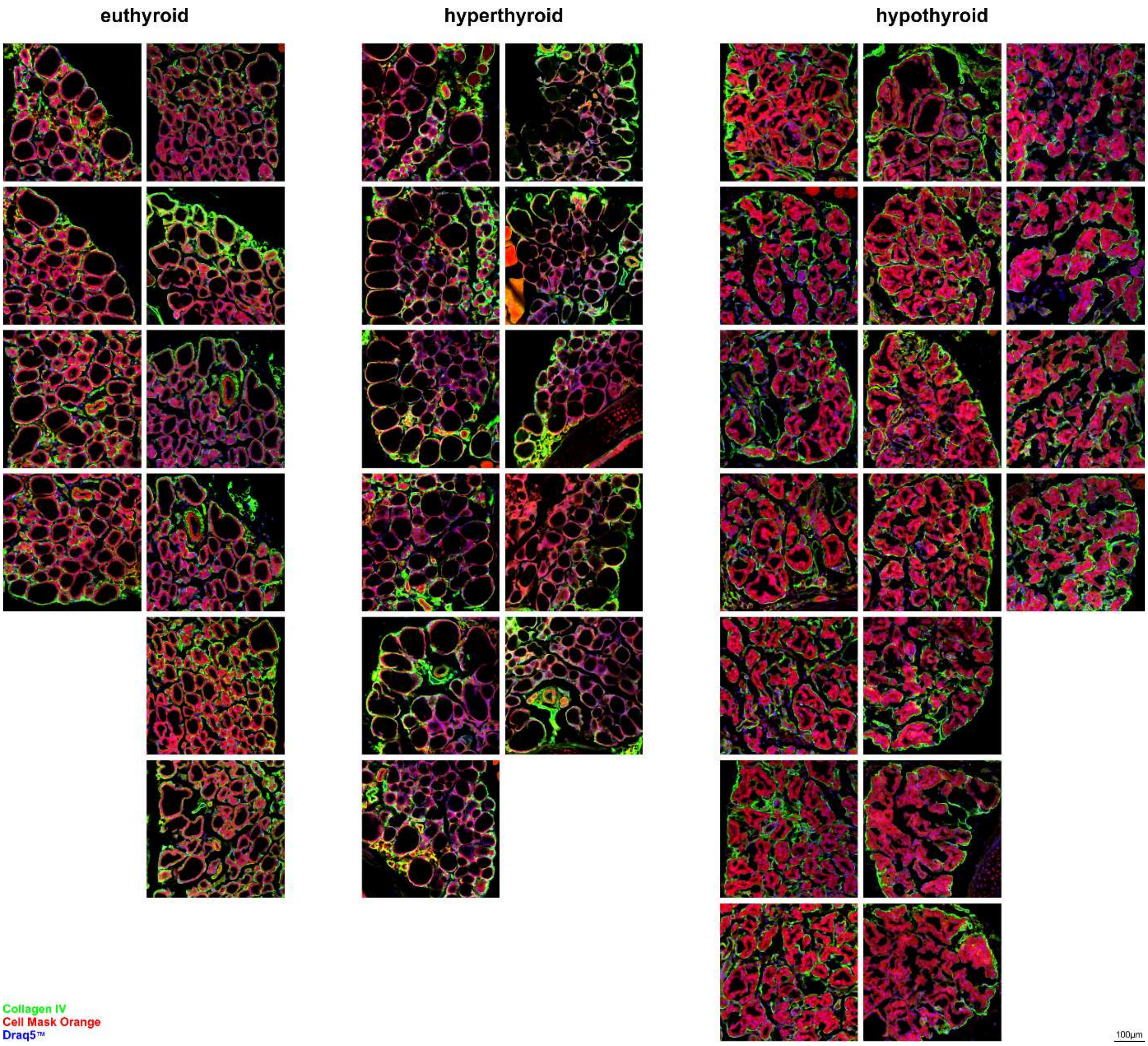

Supplementary Figure S2: Middle sections of thyroid lobes from mice of different thyroid states as indicated upon CMO staining of cytoplasm, anti-collagen IV immunostaining and Draq5™ counter-staining.

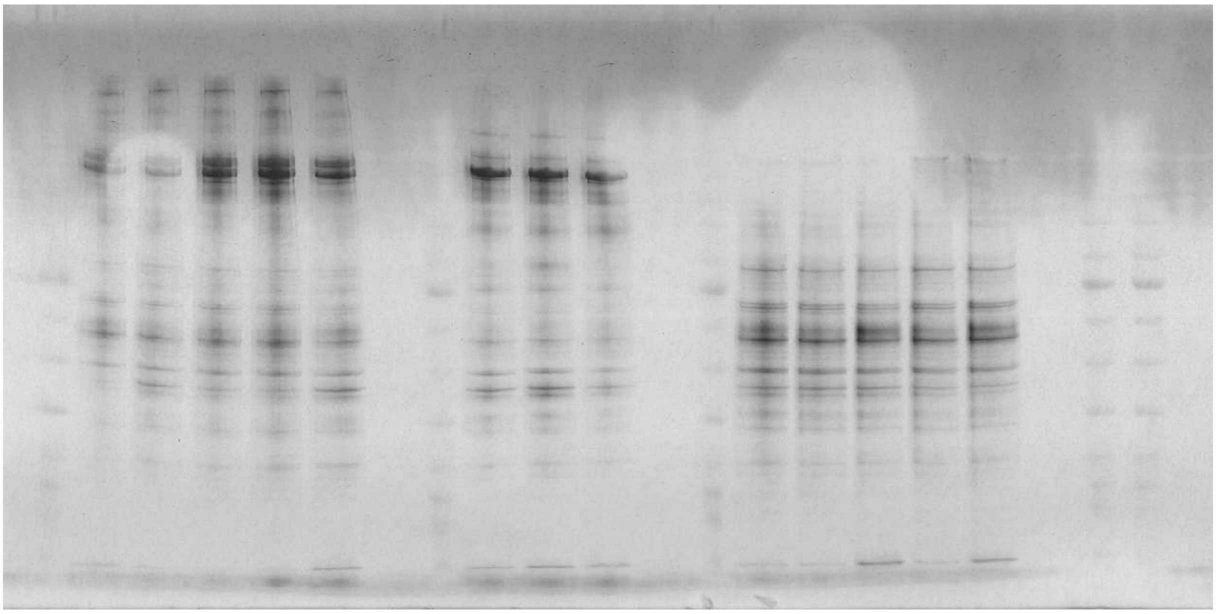

Supplementary Figure S3: SDS-gel of thyroid tissue lysates upon silver-staining.

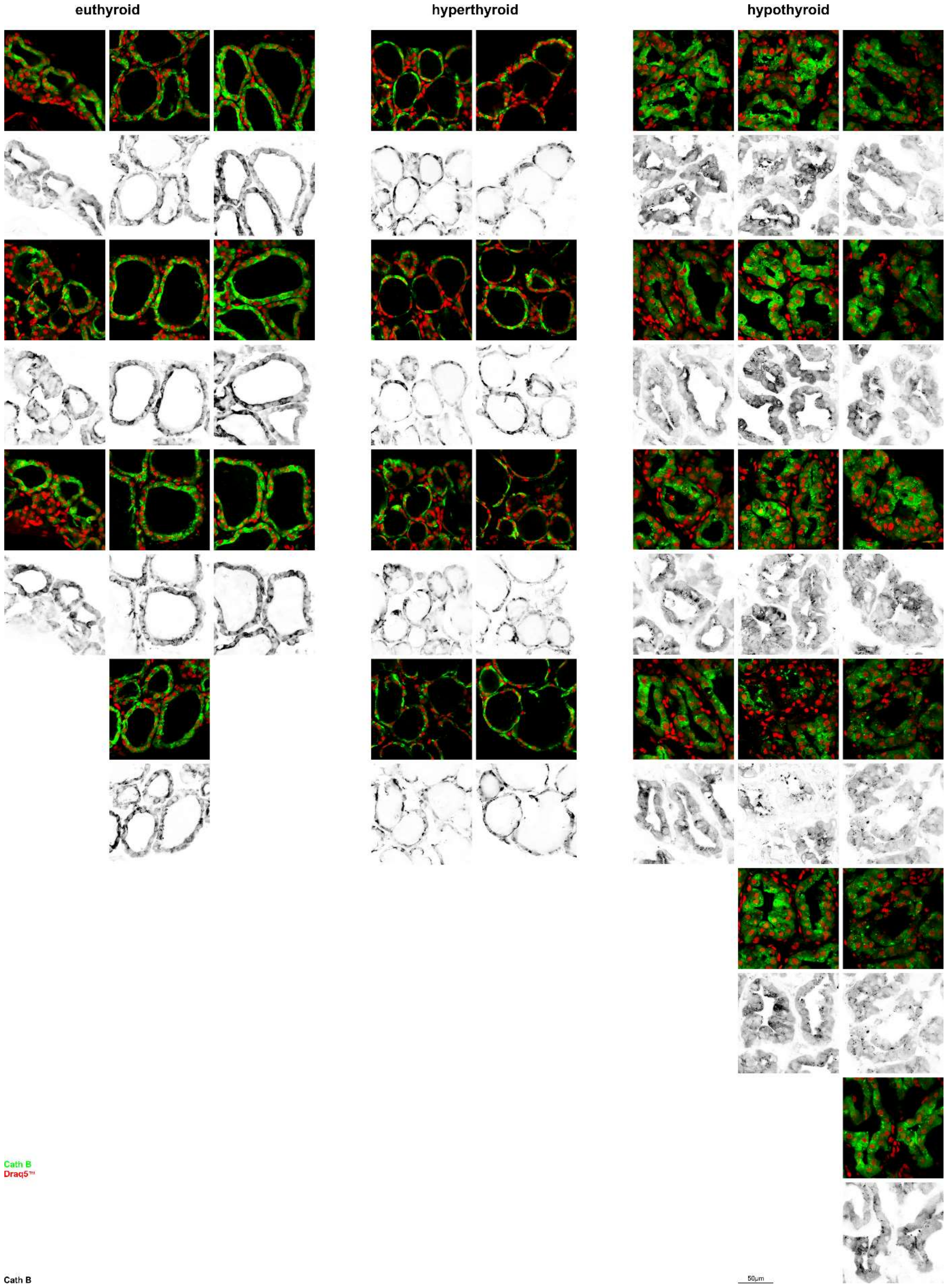

Supplementary Figure S4: Cryo-sections of thyroid tissue from mice of different thyroid states as indicated upon anti-cathepsin B immunostaining and Draq5™ counter-staining.

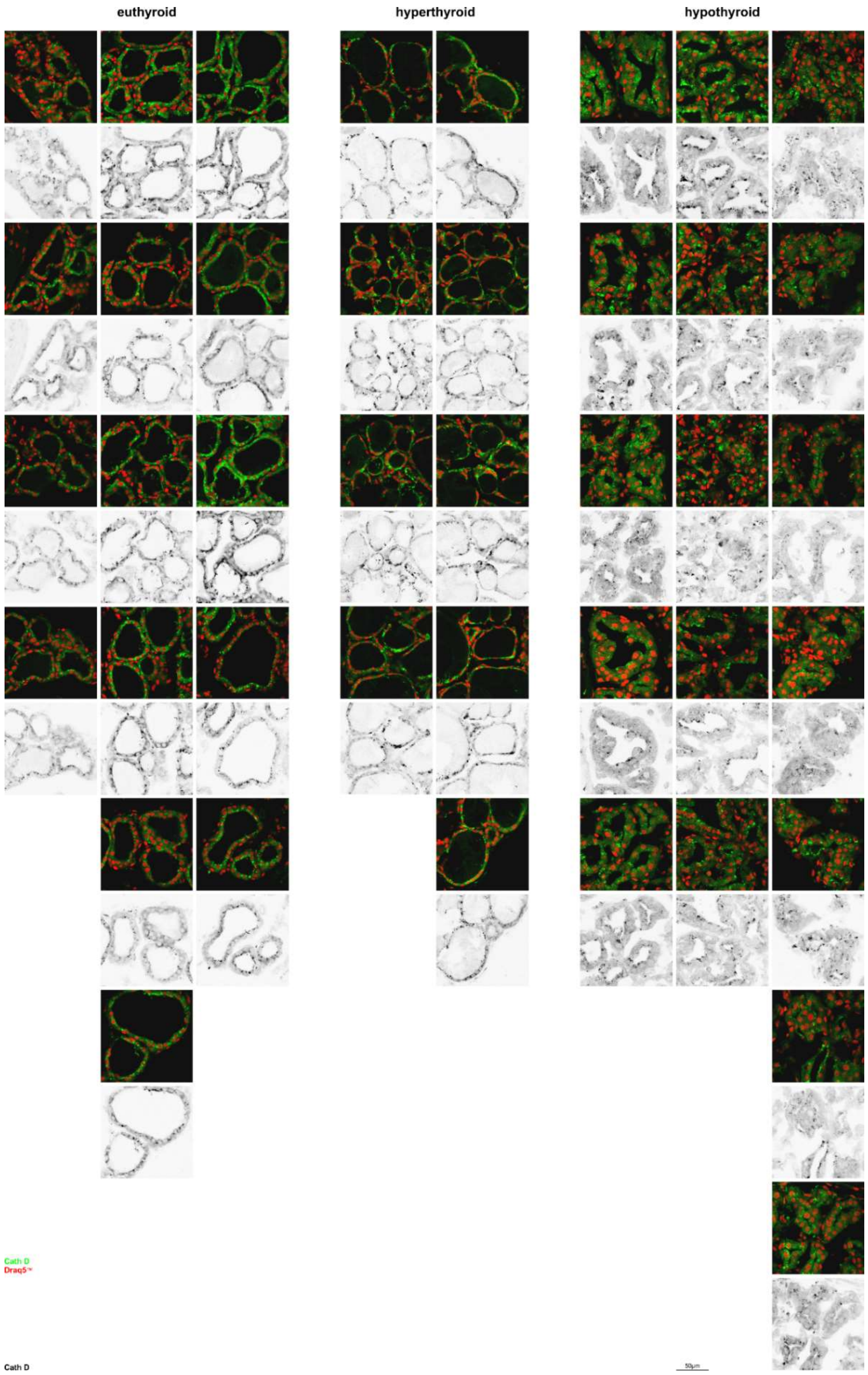

Supplementary Figure S5: Cryo-sections of thyroid tissue from mice of different thyroid states as indicated upon anti-cathepsin D immunostaining and Draq5™ counter-staining.

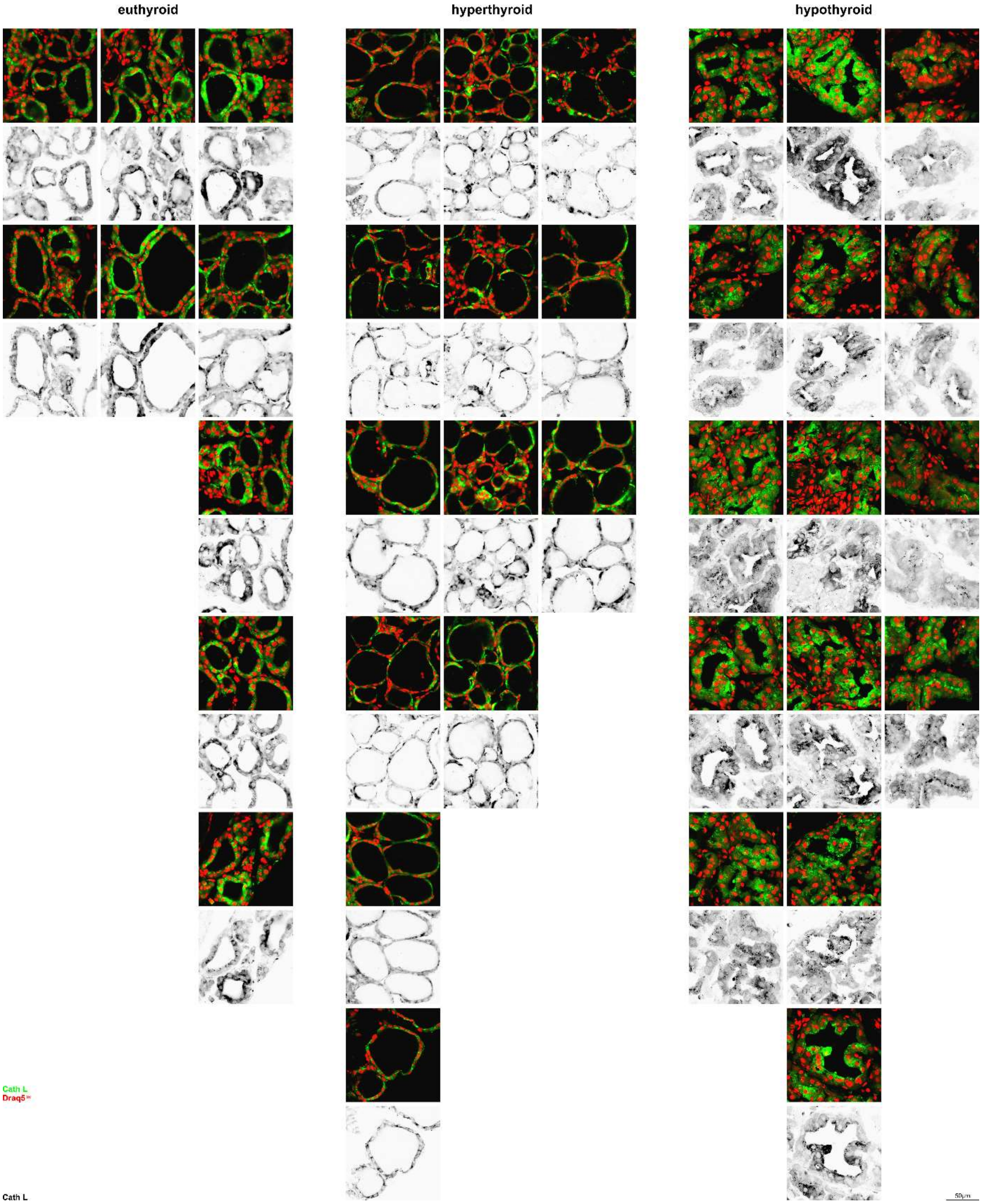

Supplementary Figure S6: Cryo-sections of thyroid tissue from mice of different thyroid states as indicated upon anti-cathepsin L immunostaining and Draq5™ counter-staining.

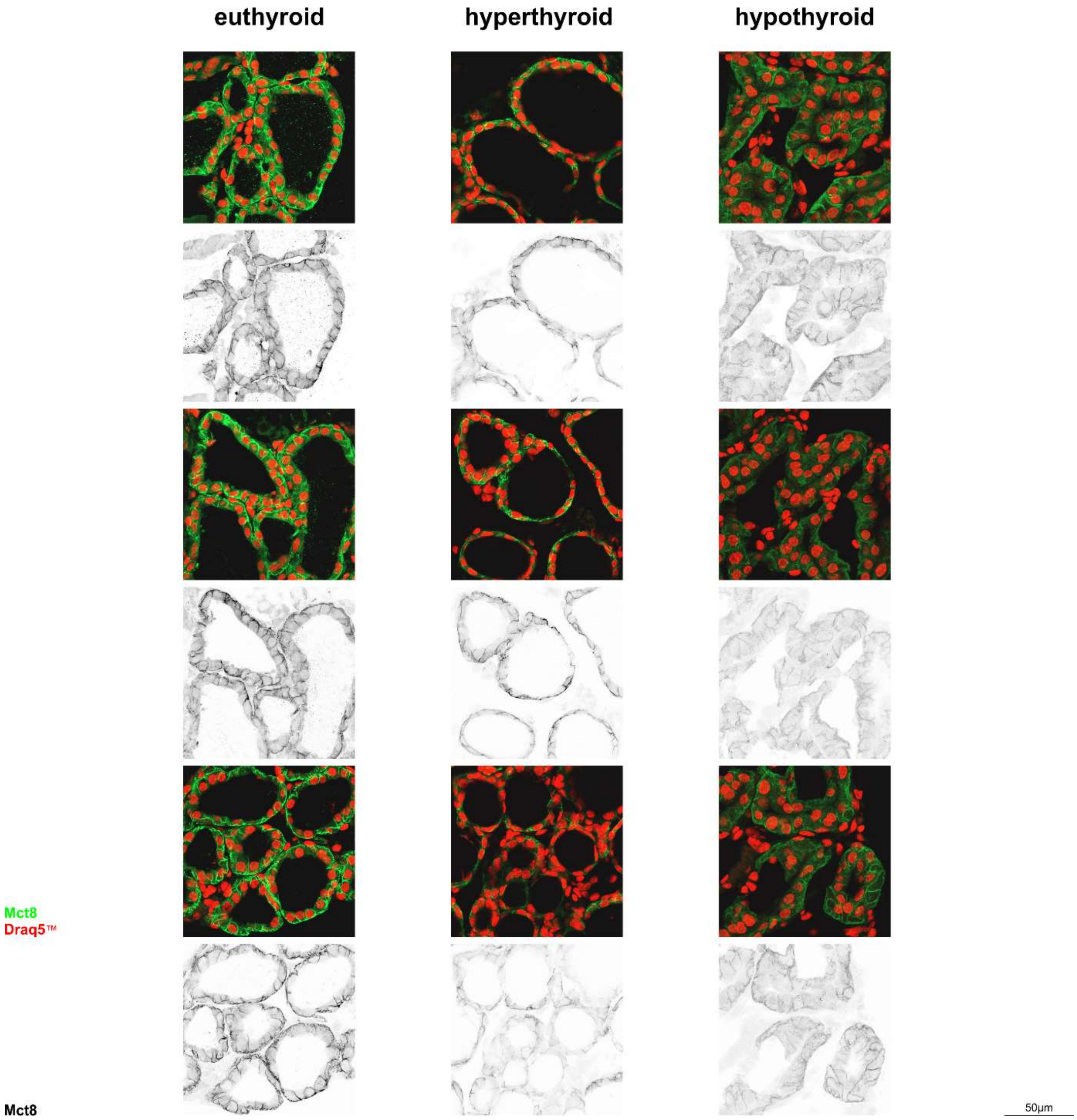

Supplementary Figure S7: Cryo-sections of thyroid tissue from mice of different thyroid states as indicated upon anti-Mct8 immunostaining and Draq5™ counter-staining.

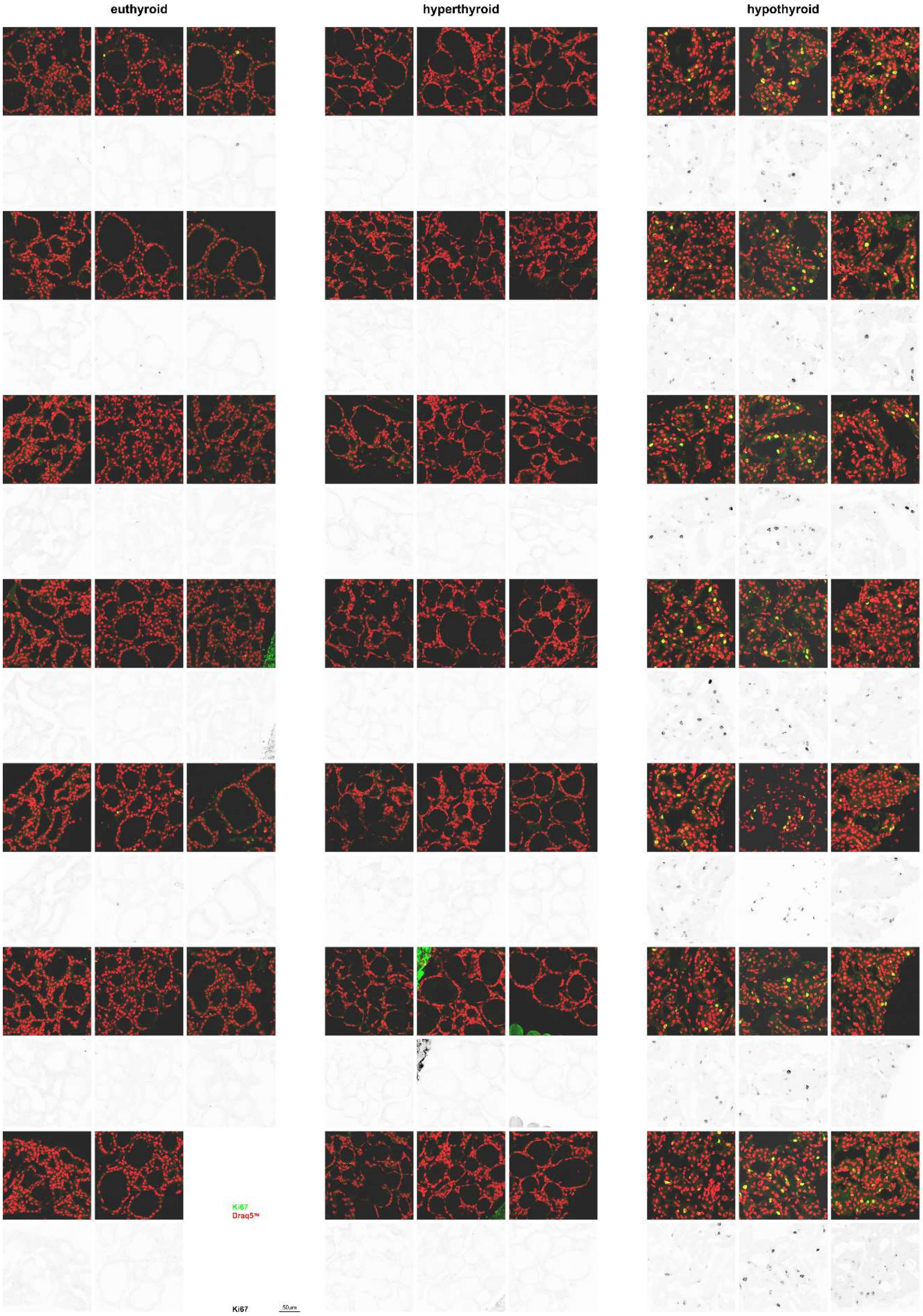

Supplementary Figure S8: Cryo-sections of thyroid tissue from mice of different thyroid states as indicated upon anti-Ki67 immunostaining and Draq5™ counter-staining.

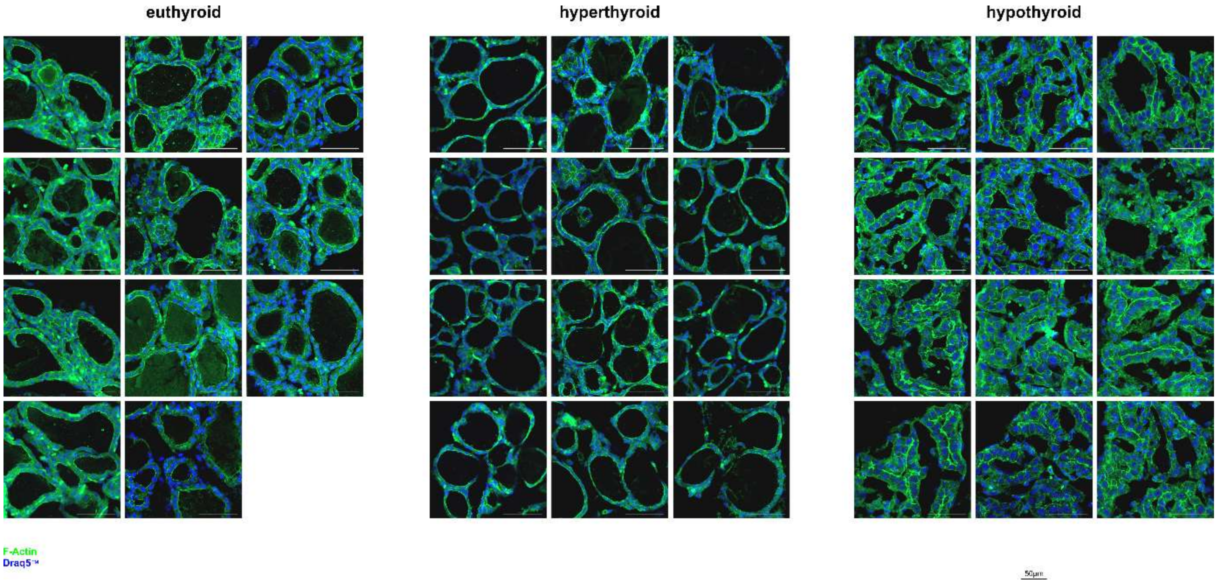

Supplementary Figure S9: Cryo-sections of thyroid tissue from mice of different thyroid states as indicated upon phalloidin staining of the F-actin cytoskeleton and Draq5™ counter-staining.

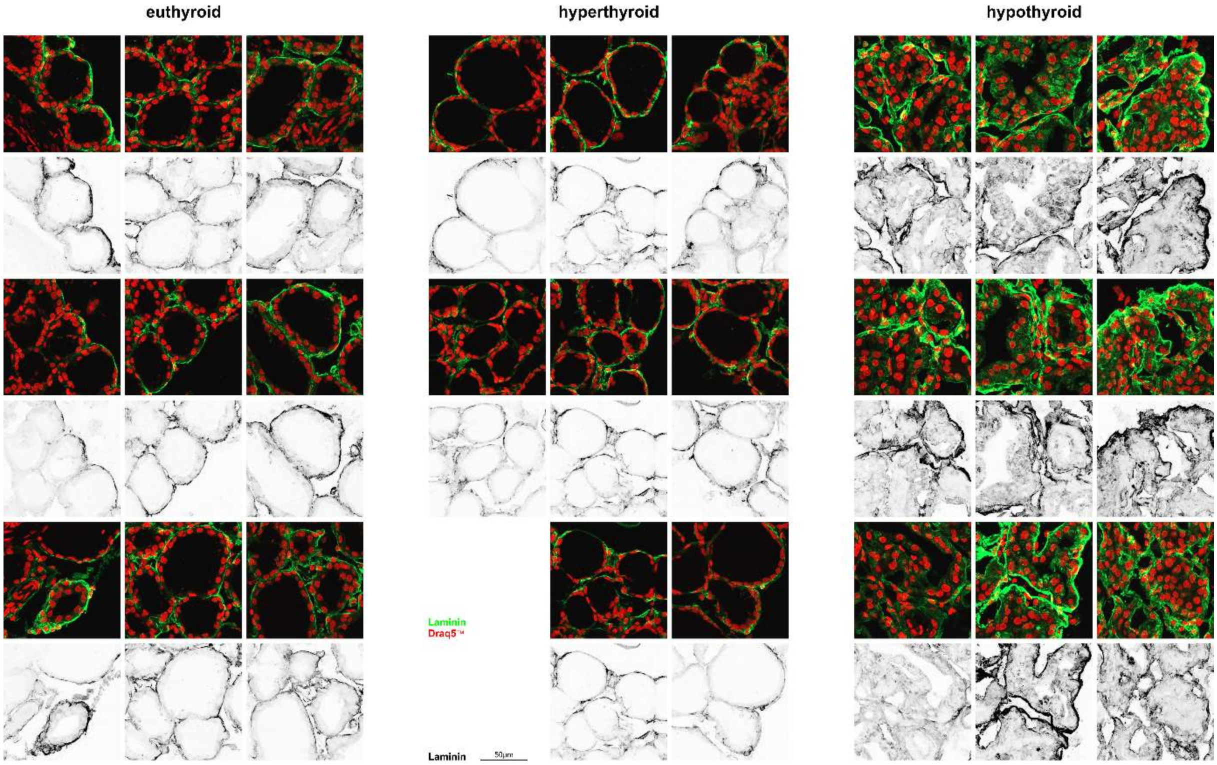

Supplementary Figure *S*10 Cryo-sections of thyroid tissue from mice of different thyroid states as indicated upon anti-laminin immunostaining and Draq5™ counter-staining.

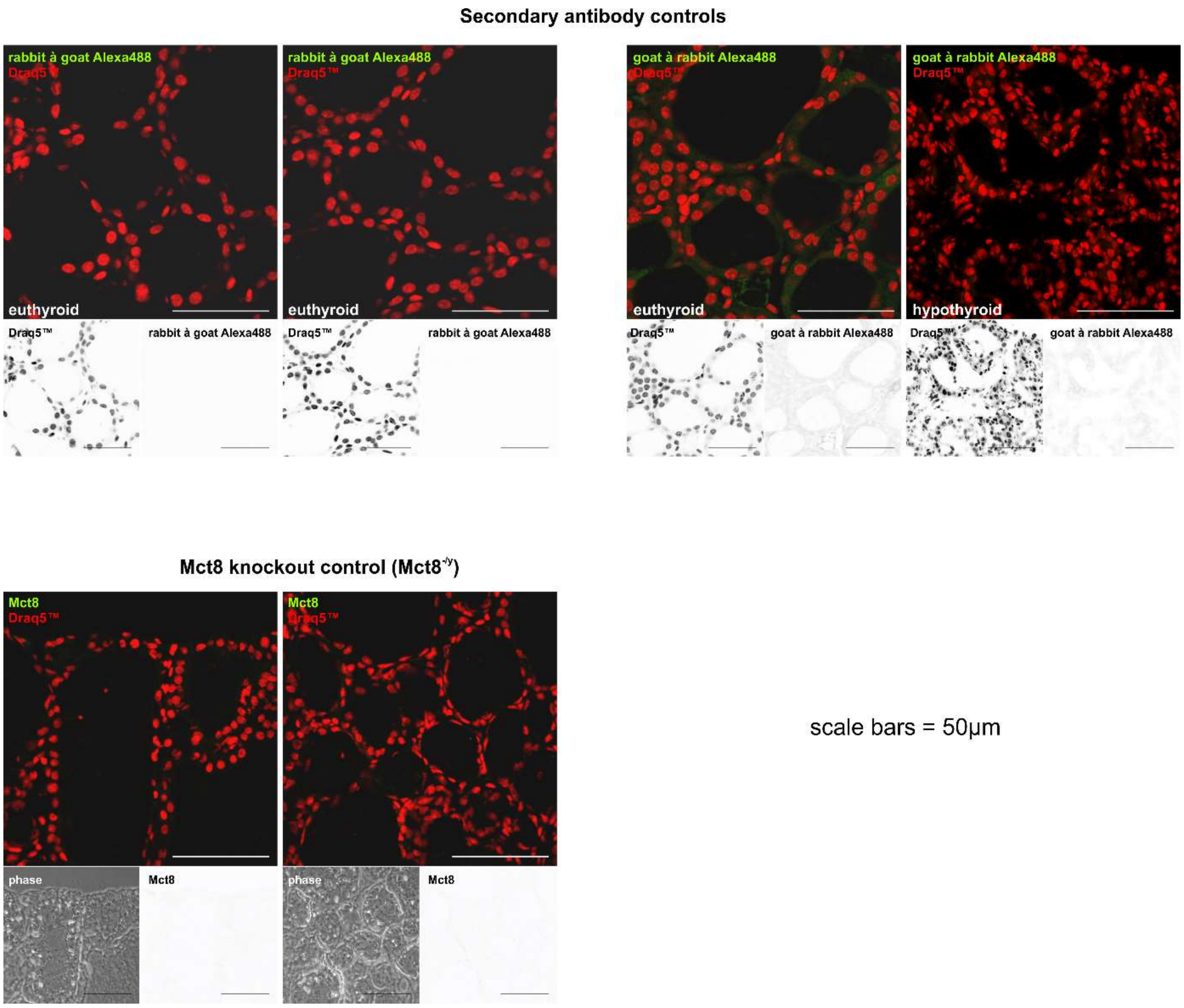

Supplementary Figure S11: Cryo-sections of thyroid tissue from mice of different thyroid states as indicated upon secondary antibody staining and Draq5™ counter-staining; and cryo-sections of thyroid tissue from Mct8-deficient mice (Mct8$^{-/y}$) as indicated serving as control of the anti-Mct8 immunostaining.
